# Language Processing as a Precursor to Language Change: Evidence From Icelandic

**DOI:** 10.3389/fpsyg.2019.03013

**Published:** 2020-01-17

**Authors:** Ina Bornkessel-Schlesewsky, Dietmar Roehm, Robert Mailhammer, Matthias Schlesewsky

**Affiliations:** ^1^Cognitive and Systems Neuroscience Research Hub, University of South Australia, Adelaide, SA, Australia; ^2^School of Psychology, Social Work and Social Policy, University of South Australia, Adelaide, SA, Australia; ^3^Centre for Cognitive Neuroscience, University of Salzburg, Salzburg, Austria; ^4^Department of Linguistics, University of Salzburg, Salzburg, Austria; ^5^School of Humanities and Communication Arts, Western Sydney University, Penrith, NSW, Australia; ^6^The MARCS Institute for Brain, Behaviour and Development, Western Sydney University, Penrith, NSW, Australia

**Keywords:** language comprehension, language change, event-related potentials, Icelandic, N400, late positivity

## Abstract

One of the main characteristics of human languages is that they are subject to fundamental changes over time. However, because of the long transitional periods involved, the internal dynamics of such changes are typically inaccessible. Here, we present a new approach to examining language change via its connection to language comprehension. By means of an EEG experiment on Icelandic, a prominent current example of a language in transition, we show that the neurophysiological responses of native speakers already reflect projected changes that are not yet apparent in their overt behavior. Neurocognitive measures thus offer a means of predicting, rather than only retracing, language change.

## 1. Introduction

Since the earliest days of the human species, human culture, and society have undergone a continuous series of changes and adaptations. Language, as the primary means of human communication, has always played an integral role in this process. English is a particularly good example of how profound such changes can be. From Old English (~400–1100 AD) to Modern English, the language has undergone at least two radical transitions: word order became fixed and the language's rich morphological system (e.g., case inflections) was drastically reduced. The communicative consequences of these changes are profound, because the properties in question crucially influence the way in which meaning can be extracted from the speech stream in real time (Bornkessel-Schlesewsky et al., 2015). In modern English, the fixed positioning of elements allows hearers to determine “who is doing what to whom” in a strictly linear manner (in transitive sentences with default verb classes, the Actor performing the action precedes the Undergoer affected by that action). In Old English or other Germanic languages such as modern German, by contrast, these relations are determined less by linear position and rather mainly by the form in which the event participants are expressed (e.g., via nominative or accusative case marking).

Between the thirteenth and fifteenth centuries (Allen, 1995), English underwent a transition from a grammar favoring morphological marking as the primary means of expressing participant roles in a sentence (“Grammar A”), to a grammar using linear position to the same purpose (“Grammar B”). The tendency toward such a change is a key property of the Germanic language family as a whole (Faarlund, 2001; Platzack, 2002).

However, the internal dynamics of the transitional processes from Grammar A to Grammar B appear virtually inaccessible, since an observation of the relevant changes (e.g., in spoken or written language) presupposes that they have already taken place in individuals. And even zooming in on the individual, the question is what exactly triggers changes in speech behavior? Theories on language change offer different perspectives on what causes change and how it begins^1^. Perception-based approaches generally assume that the perception or interpretation of input material changes in individuals, and that this is then transferred onto production. Examples for this type of perspective are speech-perception-based models of sound change (e.g., Ohala, 1981), and mainstream models of grammaticalization (e.g., Hopper and Traugott, 1993). By contrast, production-based models see changes as by-products of production (see e.g., Bybee, 2010; Harrington, 2012). For the types of changes mentioned above, i.e., changes in the way in which grammatical relations and semantic roles are indicated, both approaches are relevant, albeit to different degrees. The loss of morphological marking is a typical consequence of phonological erosion, a result of production (Bybee, 2010). In the case of English, for example, the phonological reduction and loss of unstressed final syllables is typically seen as a consequence of a fixed dynamic accent at the left edge of words. The utilization of constituent order to express grammatical relations, however, is best understood as an effect of processing information that is increasingly ambiguously marked. In fact, ambiguous structures are usually seen as the key element for triggering a reinterpretation in most theories of grammaticalization. This, in turn, leads to the recruitment of constituent ordering for purposes of expressing grammatical relations.

From the perspective of perception-based approaches to language change, then, reinterpretation by hearers precedes overt changes in how language is produced by speakers. Accordingly, overt manifestations of language change in speech or writing should be preceded by measurable preparatory changes in neural language comprehension mechanisms. We propose that this hypothesis may be investigated by measuring the brain activity of individuals who speak a language in transition. Electrophysiological measures appear particularly well-suited to revealing such effects as they (a) allow us to observe distinctions that are not consciously accessible to speakers (e.g., Bornkessel et al., 2004), and (b) have been shown to be sensitive to the transitional phenomena under examination here, namely word order and case marking (for reviews, see Bornkessel and Schlesewsky, 2006a; Bornkessel-Schlesewsky and Schlesewsky, in press). From this perspective, the relation between language processing and language change constitutes an intriguing challenge for examining the brain-behavior interface. If our assumptions are correct, it may eventually be possible to link processing phenomena at the timescale of several hundred milliseconds to cross-generational changes in language use (cf. also Christiansen et al., 2016).

Here, we take a first step toward testing the hypothesis that changes in language comprehension may precede overt language change by comparing electrophysiological correlates of sentence comprehension to judgements of sentence acceptability in Icelandic. Within the Germanic language family, Icelandic stands out for its parallels to English during the transitional period. Thus, it has a fully fledged system of morphological case marking including non-nominative subjects, but shows considerable word order strictness such as a fixed subject position (Zaenen et al., 1990; Thrainsson, 2014)^2^. In terms of linear order, Icelandic therefore behaves very similarly to Modern English (Grammar B), while its morphological properties render it more closely comparable to earlier stages of the English language (Grammar A). In addition, there are initial indications that the morphological (case) system is becoming unstable, as speakers are showing an increasing tendency to reduce the number of different case forms that can occur in particular linear positions in the sentence—a phenomenon known as “case sickness” (Smith, 1994; Eythórsson, 2000). As discussed in detail by Smith (1994), two alternations of this type “occur in most Germanic languages at some stage” (Smith, 1994, p. 675): the tendency for accusative subjects of experiencer verbs to be marked with dative (dative substitution, DS) and the tendency for accusative or dative subject marking to be replaced by nominative (nominative substitution, NS). The following Icelandic examples from Jónsson and Eythórsson (2005) illustrate DS (example 1) and NS (example 2), respectively (see their paper for further examples from Faroese and Smith, 1994 for examples from other Germanic languages such as German and Old English):


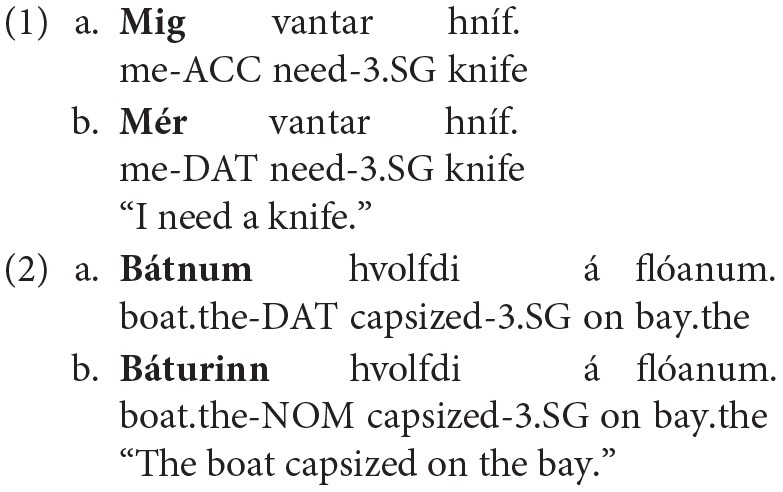


Nominative substitution parallels the diachronic changes that took place in the history of English, as a result of which the dative or accusative marking of experiencer arguments was replaced with nominative. This is illustrated by the examples in 3 (cited from Smith, 1994) using the verb *ofhreowan* (“to pity”). Both example sentences stem from the writings of lfric of Eynsham, an English abbot who lived in the tenth and eleventh centuries AD.


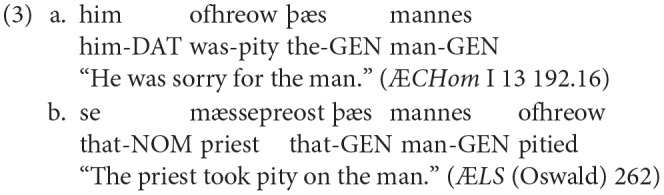


### 1.1. The Present Study

The present study used event-related potentials (ERPs) to investigate how native speakers of Icelandic process constructions that differ in regard to their compatibility with the target grammar of change (Grammar B). While we did not contrast language comprehension and production directly, we compared electrophysiological correlates of language comprehension to participants' own acceptability judgements as a first step toward a full-fledged examination of the perception-driven hypothesis of language change. Thus, we compared participants' neural responses to their overt, language-related behavior. As already discussed above, ERP responses do not directly reflect individuals' conscious assessment of sentence wellformedness (e.g., Bornkessel et al., 2003, 2004; Bornkessel and Schlesewsky, 2006b), rather mirroring the demands of online sentence processing (see also Demiral et al., 2008). This allows us to examine potential shifts in these demands vis-à-vis how participants view their own language. If overt language change has already taken place, new structures will be both produced by speakers and judged to be acceptable by hearers, even though they may not be considered grammatical from a prescriptive perspective. Here, we examine the extent to which neural language processing and acceptability judgements differ from (prescriptive) grammatical assumptions and whether this comparison can shed new light on the dynamics of language change.

Participants were presented with two critical types of sentences (see Table 1): structures with an initial dative and a post-verbal nominative and structures with an initial nominative and a post-verbal dative. We assume that the nominative-before-dative sequence is the target structure on which a fully completed transition to (the Modern English type) Grammar B will finally converge. We can thus use the differential brain response to these structures as opposed to their dative-before-nominative counterparts as a diagnostic tool for how far the neural transition toward Grammar B has advanced.

**Table 1:** Example sentences for the present study.

**Verb**	**NP2 case**	**Example**
ACT	DAT	… drekkt / **fisksalanum** / í brunninum. … *drowned fish-salesman-DAT in well-the*
	NOM	*… drekkt / **fisksalinn** / í brunninum. … *drowned fish-salesman-NOM in well-the*
ALT	DAT	… fylgt / **konunni** / í borginni. … *followed lady-DAT in city-the*
	NOM	… fylgt / **konan** / í borginni. … *followed lady-NOM in city-the*
EXP	DAT	*… mislíkað/ **nemandanum** / á kaffihúsi. … *disliked student-DAT in coffeehouse-the*
	NOM	… mislíkað/ **nemandinn** / á kaffihúsi. … *disliked student-NOM in coffeehouse-the*

*Note that all sentences commenced with a main clause that was common to each item, e.g., Ég vantreysti sjómanninum / sem / hefur …, I distrust seaman-the-DAT who has…, 'I distrust the seaman who has …' The critical position (NP2 in the relative clause) is marked in bold and segmentation for visual presentation is indicated by the forward slashes (/)*.

In order to characterize the degree of transition more closely, we used three verb types which differ in their compatibility between Grammar A and Grammar B: (a) active verbs, which were already associated with a nominative-before-dative structure in Grammar A and thus do not require a change to be compatible with Grammar B; (b) dative subject-experiencer verbs, which are obligatorily associated with a dative-before-nominative structure in Grammar A (and current Icelandic) and must thus undergo a transition to nominative-before-dative to be compatible with Grammar B; and (c) alternating verbs, which are already in transition between Grammar A and Grammar B in that they allow both a nominative-before-dative and a dative-before-nominative order (Barðdal, 2001).

Note that, strictly speaking, the different verb types used here are in fact associated with changes in subject case rather than just word order. Dative subject-experiencer verbs require a dative subject and nominative object. Alternating verbs, by contrast, are compatible with a nominative subject and dative object as well as with a dative subject and nominative object. Finally, active verbs require a nominative subject and dative object.

The relative clause constructions used here served to create a fixed subject-before-object word order. The subject is expressed by the relative pronoun *sem*, which is coreferent with the noun in the main clause. As *sem* is invariant across different cases, it does not become clear until the post-verbal noun in the relative clause (NP2) whether the word order is nominative-before-dative or dative-before-nominative. As the verb has already been processed at this point, NP2 is the critical position for observing expectation mismatches in regard to the word order/case marking. Based on previous ERP experiments that examined case marking and word order in several languages including German, Swedish, Japanese, and Hindi, we expect such mismatches to be reflected in an N400 followed by a late positivity (e.g., Frisch and Schlesewsky, 2001; Bornkessel et al., 2004; Mueller et al., 2005; Haupt et al., 2008; Choudhary et al., 2009; Hörberg et al., 2013). We will return to our proposed functional interpretation of these components—and how this may relate to language change—in the discussion section.

## 2. Methods

### 2.1. Participants

Twenty-three students from the University of Iceland (Reykjavik) participated in the experiment [13 female, mean age 25.39 (sd = 3.71) years, age range 17–30 years]. All participants were right-handed native speakers of Icelandic with normal or corrected-to-normal vision and gave written informed consent before the experimental session. Seven additional participants were excluded from the final data analysis due to varying numbers of trials per condition and a different task setup (acceptability task only): these were the first seven participants run, on the basis of which we concluded that the experimental protocol was too long and that a second task was required in order to avoid strategic effects.

### 2.2. Materials

Each sentence consisted of a matrix clause with a first person nominative-subject (é*g* “I”) and one of four nominative-subject experiencer verbs (*vantreysti* “distrust”; *treysti* “trust”; *man eftir* “remember”; *trúi* “believe”), which were distributed equally across conditions and were followed by a dative case marked noun (object) and a subsequent subject relative clause relating to it. The subject relative clause began with the inflexible relative pronoun *sem*, which is fully case ambiguous (NOM/DAT/GEN/ACC). Note that, in contrast to English, the relative pronoun sem must always be at the beginning of a clause and can never be preceded by a preposition (e.g., *húsið, sem hann bjó í* “The house that he lived in”). In addition, the relative pronoun cannot be dropped from the clause as in English (a general restriction in other Germanic languages). A finite auxiliary and the main verb followed the relative pronoun, thereby explicitly indicating that *sem* refers to the subject of the relative clause. After the main verb of the relative clause, there was a case-marked noun followed either by a temporal, local, reason, or manner adverbial. The type of verb within the relative clause was manipulated according to the design in Table 1: active verbs, alternating verbs, and dative-subject experiencer verbs (the choice of verbs was motivated by Barðdal, 2001; Jónsson, 2003). For each verb type, two different sentence types were created: the post-verbal noun was either marked with dative or nominative case marking. Participants read 48 sentences in each of the two conditions with active verbs, 24 sentences in each of the two conditions with alternating verbs, and 20 sentences in each of the conditions with dative-subject experiences verbs, thus resulting in a total of 184 sentences. The differing trial numbers between verb classes were chosen so as to ensure an equal split between default (active) and non-default (alternating, dative-subject experiencer) verbs. Sentences were presented to participants in a pseudo-randomized manner.

### 2.3. Procedure

Sentences were presented visually in the center of a computer screen. Each trial began with the presentation of an asterisk (1,000 ms) in order to fixate participants' eyes at the center of the screen and to alert them to the upcoming presentation of the sentence. Main clauses were presented as a single chunk (1,000 ms), followed by a word-by-word presentation of the relative clause. Each word was presented for 750 ms (adverbials were presented as chunks), with an inter-stimulus interval (ISI) of 150 ms. This relatively long presentation time was chosen because of the morphological complexity of the language (for similar arguments for Turkish, see Demiral et al., 2008) and was perceived as a comfortable reading rate by participants. After the presentation of the sentence, there was a 400 ms pause before participants were required to complete an acceptability judgment task (signaled through the presentation of a question mark), which involved judging whether the sentence was acceptable or not. Participants responded by pressing the left or right mouse button for “yes” or “no.” The time window for the button press was restricted to 3,000 ms. Afterwards, participants responded to a comprehension question (an indirect interrogative sentence querying actor/undergoer roles). Again, the maximal reaction time for this task was 3,000 ms. Trials were separated by an inter-trial interval (ITI) of 1,250 ms.

Participants were asked to avoid movements and eye-blinks during the presentation of the sentences. All experimental sessions began with a short training session followed by 8 experimental blocks, between which the participants took short breaks. Each experimental session lasted ~2 h (including electrode preparation).

### 2.4. EEG Recording and Preprocessing

The EEG was recorded by means of 29 sintered Ag/AgCl-electrodes fixed at the scalp by means of an elastic cap (Easy Cap, Herrsching-Breitbrunn, Germany). The ground electrode was positioned at C2. Recordings were referenced to the left mastoid. The electrooculogram (EOG) was monitored by means of electrodes placed at the outer canthus of each eye for the horizontal EOG and above and below the participant's left eye for the vertical EOG. Electrode impedances were kept below 5 kOhm. All EEG and EOG channels were amplified using a BrainVision BrainAmp amplifier (time constant 10 s, high cutoff 250 Hz) and recorded with a digitization rate of 500 Hz.

EEG data were preprocessed using MNE Python version 0.19.1 (Gramfort et al., 2013, 2014) supplemented by additional utility functions from the philistine package (https://gitlab.com/palday/philistine). EOG artifacts were corrected using Independent Component Analysis (ICA). To this end, a copy of the raw data was bandpass filtered from 1 to 40 Hz (zero-phase, hamming windowed FIR filter; length: 1,651 samples; transition bandwidth: 1–10 Hz). ICAs were computed using the FastICA method with 25 components (EEG channels only; epochs with peak-to-peak voltages exceeding 250 microvolts were excluded from consideration). We used the “create_eog_epochs” function in MNE to find EOG events; these were then used to identify EOG-related ICs via correlation (function “ica.find_bads_eog”). The components thus identified were removed from the original raw data. Subsequently, the data were filtered with a 0.1–30 Hz bandpass filter (zero-phase, hamming windowed FIR filter; filter length: 16,501 samples; transition bandwidth: 0.1–7.5 Hz) to exclude slow signal drifts and high frequency noise. The data were epoched from –200 to 1,200 ms relative to the onset of the critical second NP. Epochs with peak-to-peak amplitudes exceeding 150 microvolts for EEG channels were excluded, as were flatlining epochs with peak-to-peak voltages under 5 microvolts. No baseline correction was applied; rather, the trial-by-trial mean prestimulus voltage (–200 to 0 ms) was included as a covariate in the statistical analysis and used to baseline-correct the plots (Alday, 2019).

### 2.5. Data Analysis

We used R Version 3.6.1 for all statistical analyses (R Core Team, 2018) and the packages tidyverse version 1.2.1 (Wickham et al., 2019), lme4 version 1.1.21 (Bates et al., 2015), car version 3.0-4 (Fox and Weisberg, 2011), emmeans version 1.4.2 (Lenth, 2019), and cowplot version 1.0.0 (Wilke, 2019). Raincloud plots were produced using the method and code supplied by Allen et al. (2019). To produce model output tables, we used lmerOut version 0.5 (Alday, 2018) and kableExtra version 1.1.0 (Zhu, 2019). Raw data and all analysis scripts are available via the Open Science Framework (see Data Availability Statement.)

For all analyses below, contrasts for categorical factors used sum coding (for a tutorial on contrast coding, see Schad et al., 2020), i.e., coefficients reflect differences to the grand mean.

#### 2.5.1. Behavioral Data

Behavioral data were analyzed using generalized mixed effects models with fixed effects verb and case and random intercepts by participant and item. More complex random effect structures involving random slopes by participant and item did not converge.

#### 2.5.2. EEG Data

Single-trial EEG data were analyzed using mixed effects models with fixed effects verb, case, and epoch (i.e., time within the experiment), topographical factors laterality and sagittality and their interaction. Laterality and sagittality were implemented as continuous predictors so as to provide a more fine-grained perspective on topographical similarities and differences between individual electrodes (see Brilmayer et al., 2019). To this end, we used positional coordinates retrieved from http://robertoostenveld.nl/electrodes/besa_81.txt. We standardly include epoch as a fixed effect when analysing EEG data in order to examine whether effects change over the course of the experiment. Individual trial mean prestimulus EEG amplitude (–200 to 0 ms) was included in the model as a covariate in lieu of baseline correction (Alday, 2019). (See also Alday and Kretzschmar, 2019, for an example of this approach). Epoch and prestimulus EEG amplitude were centered prior to their inclusion in each model. Models also included random slopes for the interaction of verb and case by participant and for case by item. More complex random effects structures including trial led to convergence problems. We analyzed single-trial ERP amplitudes in the following two time windows: 300–500 ms for the N400 and 700–1,000 ms for the late positivity.

## 3. Results

### 3.1. Behavioral Data

**Figure 1:**
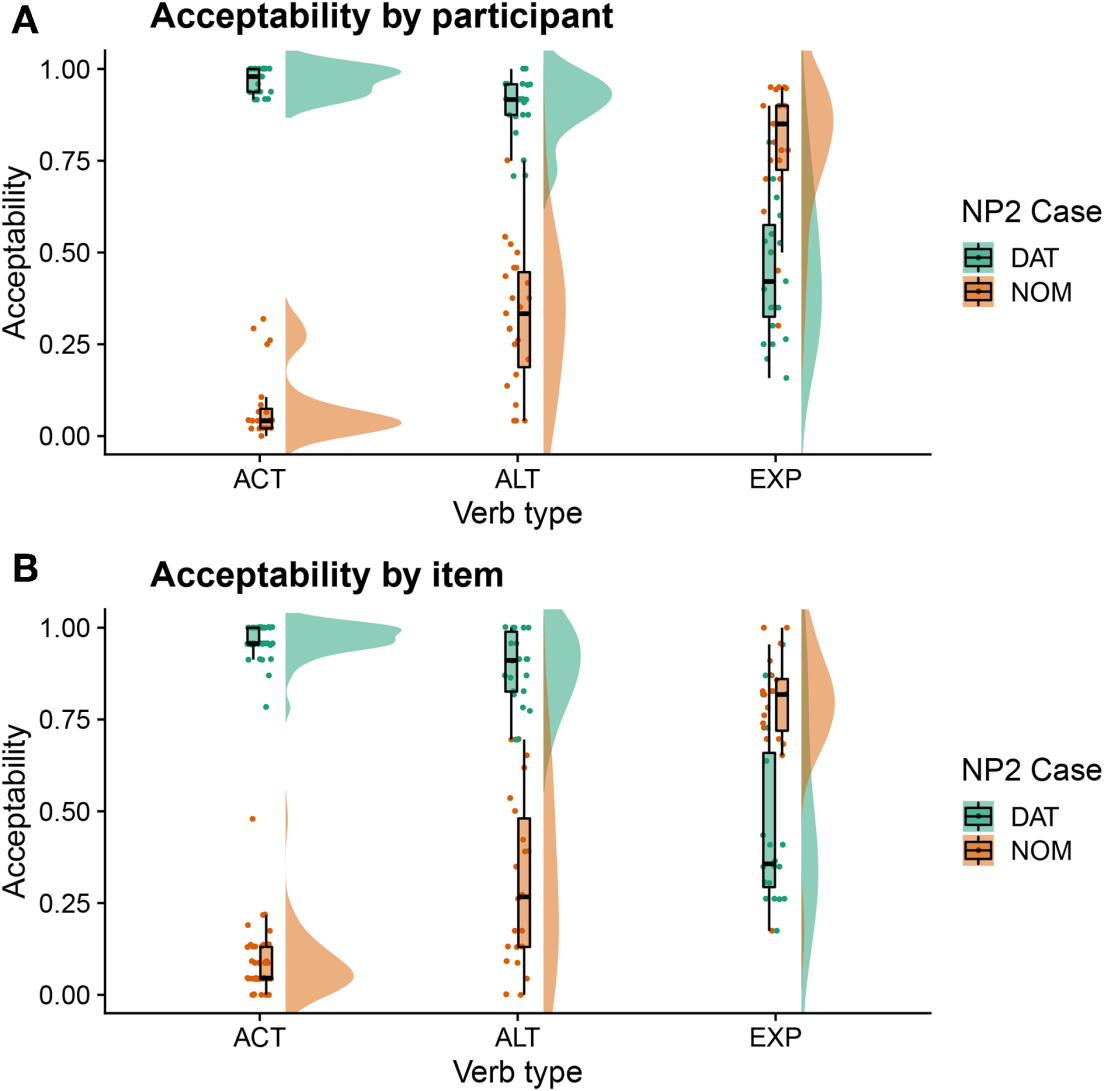
Acceptability judgements including by-participant **(A)** and by-item **(B)** variability. Individual data points represent the mean by-participant/by-item acceptability of the verb and case combination.

The results of the acceptability judgement task are visualized in Figure 1 using raincloud plots (Allen et al., 2019). Figure 1A shows variability by participant, i.e., individual data points represent the mean by-participant acceptability of each verb and case combination. Figure 1B, by contrast, shows variability by item, i.e., individual data points represent the mean by-item acceptability of each verb and case combination. As is apparent from the figure, active verbs showed a clear preference for a dative-marked NP2, i.e., for nominative-dative orders. This was the case both by participants and items. Alternating verbs also showed a general preference for nominative-dative orders, but with a less clear-cut pattern than active verbs. While nominative-dative orders were highly acceptable for all participants and items, there was considerably more variability for dative-nominative orders. Finally, experiencer verbs showed an overall preference for dative-nominative orders. However, there was again considerable variability underlying this pattern. Participants varied widely with regard to how acceptable they found both orders, i.e., some participants accepted the—supposedly ungrammatical—nominative-dative order and some tended to reject the dative-nominative order. A similar pattern emerged by item.

**Figure 2:**
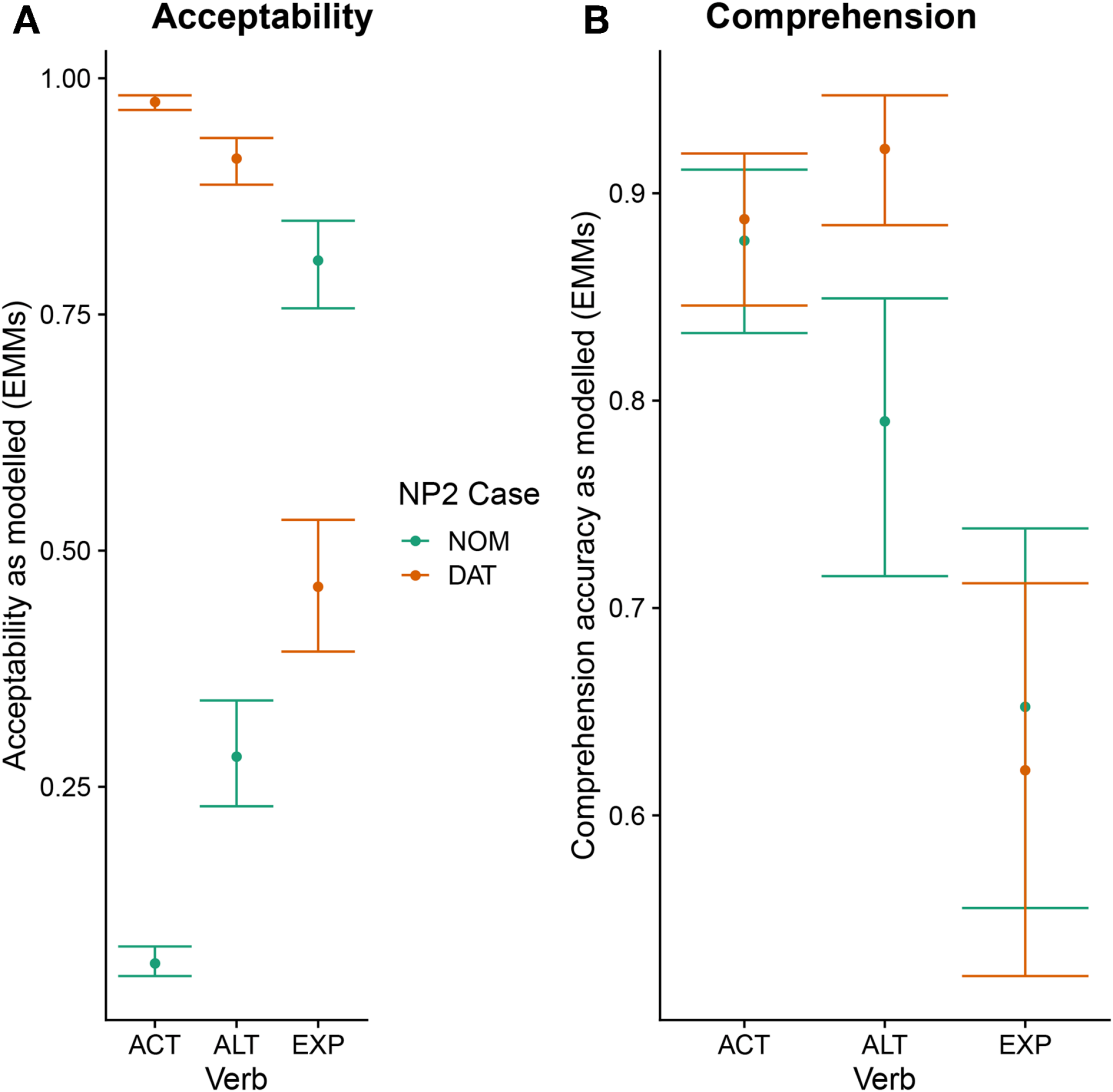
Estimated marginal means for the fitted acceptability judgement responses **(A)** and fitted comprehension question responses **(B)**. Errorbars correspond to 83% confidence intervals.

Statistical analysis of the acceptability data using generalized linear mixed effects modeling revealed main effects of verb [type II Wald test: χ^2^(2) = 13.97, *p* < 0.001] and case [χ^2^(1) = 245.57, *p* < 0.001], as well as an interaction between the two [χ^2^(2) = 856.31, *p* < 0.001]. Model estimates are visualized in Figure 2A using estimated marginal means. This also serves to resolve the interaction. The errorbars in this and the following figures represent 83% confidence intervals, the non-overlap of which corresponds to significance at the 5% level.

For the comprehension task, participants had a mean accuracy of 75% (sd: 22%). Generalized linear mixed effects modeling again showed main effects of verb [type II Wald test: χ^2^(2) = 46.51, *p* < 0.001] and case [χ^2^(1) = 9.16, *p* < 0.01], as well as an interaction between the two [χ^2^(2) = 31.37, *p* < 0.001]. Model estimates are visualized in Figure 2B using estimated marginal means. As is apparent from the figure, participants showed a high comprehension accuracy for both word orders with active verbs. In sentences with alternating verbs, by contrast, comprehension was significantly more accurate for nominative-dative than for dative-nominative orders. Finally, for the experiencer verbs, comprehension accuracy was relatively low for both word orders.

Full model summaries for the behavioral data are presented in Tables S1, S2.

### 3.2. ERP Data

**Figure 3:**
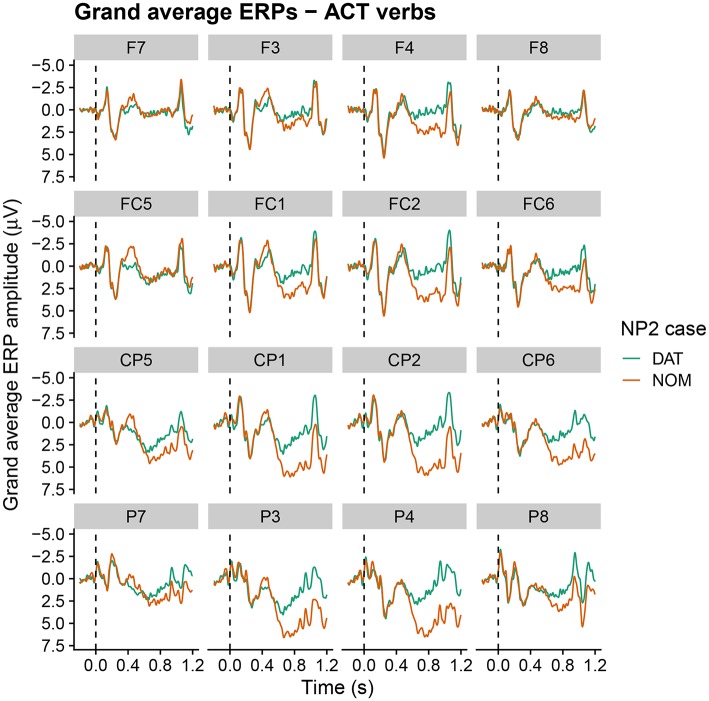
Grand average ERPs at the critical NP2 position for sentences with active verbs (onset at the dashed vertical line). Negativity is plotted upwards.

**Figure 4:**
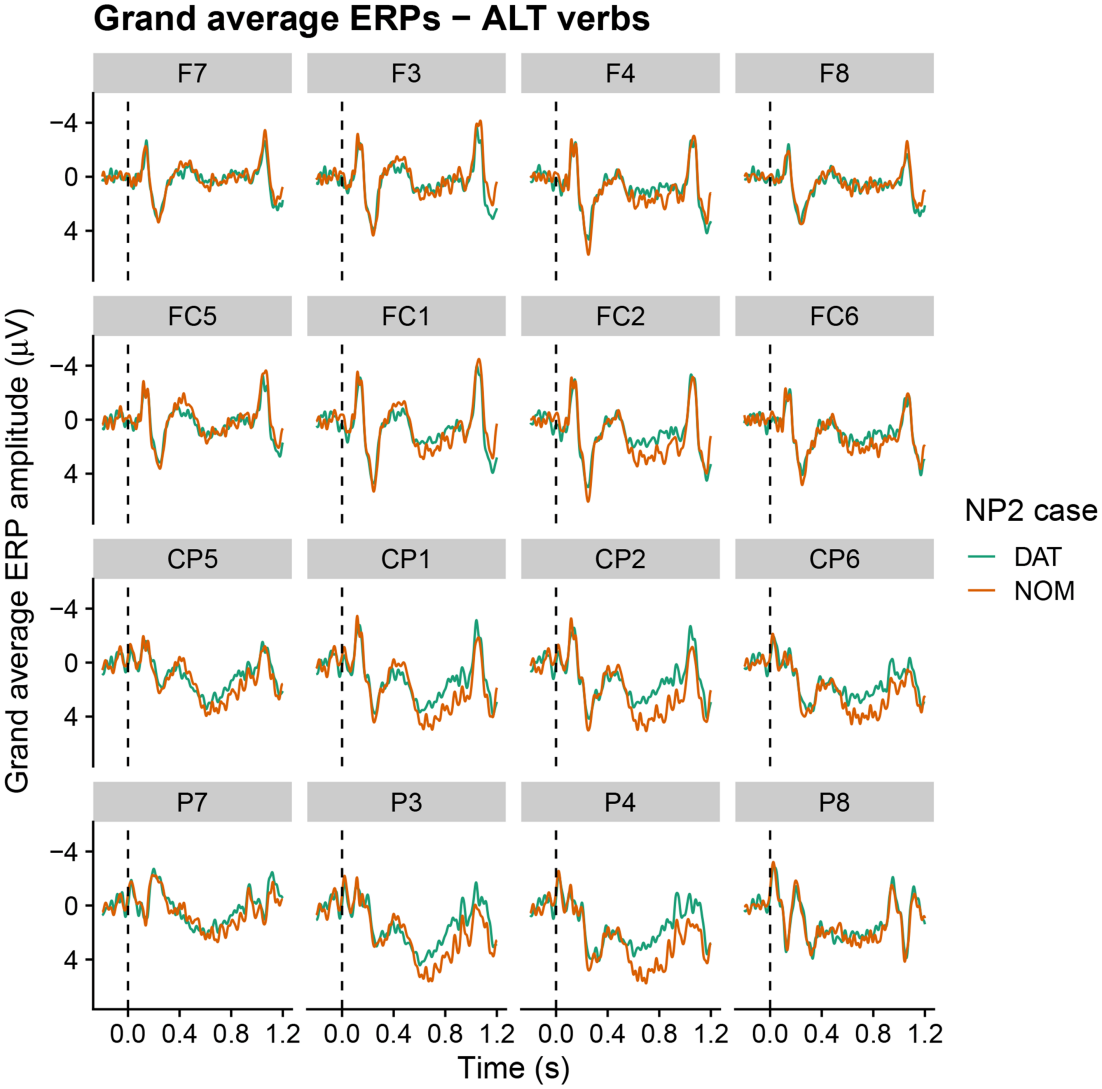
Grand average ERPs at the critical NP2 position for sentences with alternating verbs (onset at the dashed vertical line). Negativity is plotted upwards.

**Figure 5:**
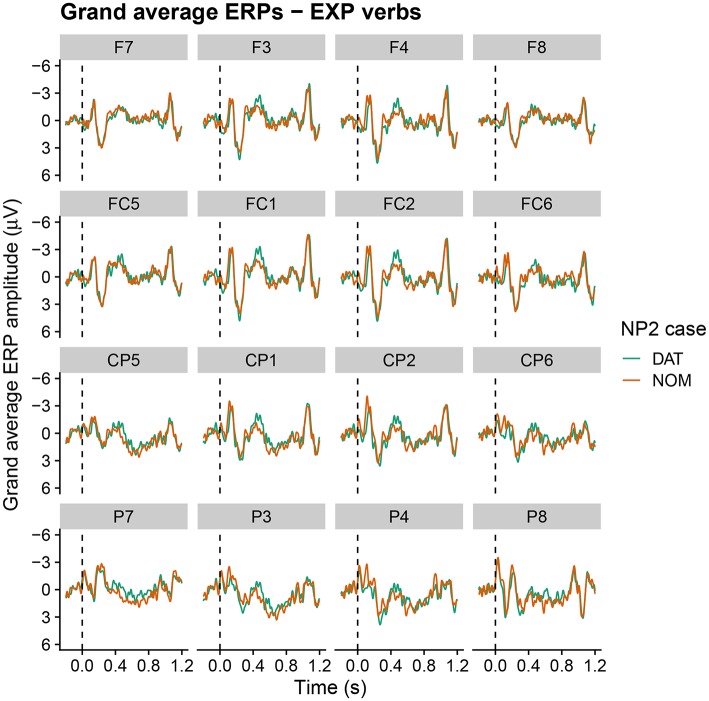
Grand average ERPs at the critical NP2 position for sentences with experiencer verbs (onset at the dashed vertical line). Negativity is plotted upwards.

Grand average ERPs at the critical NP2 position within the relative clause are shown in Figures 3–5 for active, alternating and experiencer verbs, respectively. Active verbs show a biphasic N400–late positivity pattern for dative-nominative vs. nominative-dative orders (i.e., for sentences in which NP2 is marked nominative as opposed to dative). A similar but less pronounced pattern is observable for the alternating verbs. Experiencer verbs, by contrast, show a slight tendency for a reversed pattern in the N400 (i.e., increased negativity for nominative-dative vs. dative-nominative orders), but there is no indication of differences in the late positivity. By-participant and by-item variability in the ERPs are visualized in Figures S1–S6. These show that variability by both participants and items is higher for alternating and experiencer verbs in comparison to active verbs.

The ERP data were analyzed using linear mixed effects models as outlined above Tables 2, 3 provide a broad summary of effects in the N400 and late positivity time windows, respectively, using Type II Wald tests. Full model summaries are presented in Tables S3–S6. In line with our hypotheses, we focus on interactions of verb type and case and, for each statistical model, interpret the highest-order interaction involving both of these factors.

**Table 2:** Summary of effects in N400 time window (Type II Wald Tests).

	**Chisq**	**Df**	**Pr(>Chisq)**
scale(prestim)	2643.183	1	0.000
verb	10.934	2	0.004
case	14.302	1	0.000
lat.	245.452	1	0.000
sag.	694.042	1	0.000
scale(epoch)	7.140	1	0.008
scale(prestim):verb	93.725	2	0.000
scale(prestim):case	8.088	1	0.004
verb:case	9.224	2	0.010
scale(prestim):lat.	23.091	1	0.000
verb:lat.	1.233	2	0.540
case:lat.	5.586	1	0.018
scale(prestim):sag.	107.633	1	0.000
verb:sag.	46.735	2	0.000
case:sag.	62.627	1	0.000
lat.:sag.	2.973	1	0.085
scale(prestim):scale(epoch)	22.067	1	0.000
verb:scale(epoch)	17.447	2	0.000
case:scale(epoch)	5.012	1	0.025
lat.:scale(epoch)	2.632	1	0.105
sag.:scale(epoch)	23.460	1	0.000
scale(prestim):verb:case	4.781	2	0.092
scale(prestim):verb:lat.	0.549	2	0.760
scale(prestim):case:lat.	6.555	1	0.010
verb:case:lat.	1.459	2	0.482
scale(prestim):verb:sag.	2.466	2	0.291
scale(prestim):case:sag.	15.001	1	0.000
verb:case:sag.	37.054	2	0.000
scale(prestim):lat.:sag.	0.576	1	0.448
verb:lat.:sag.	0.018	2	0.991
case:lat.:sag.	0.008	1	0.931
scale(prestim):verb:scale(epoch)	17.295	2	0.000
scale(prestim):case:scale(epoch)	2.388	1	0.122
verb:case:scale(epoch)	6.376	2	0.041
scale(prestim):lat.:scale(epoch)	0.164	1	0.685
verb:lat.:scale(epoch)	0.039	2	0.981
case:lat.:scale(epoch)	0.761	1	0.383
scale(prestim):sag.:scale(epoch)	11.177	1	0.001
verb:sag.:scale(epoch)	1.297	2	0.523
case:sag.:scale(epoch)	0.687	1	0.407
lat.:sag.:scale(epoch)	0.112	1	0.737
scale(prestim):verb:case:lat.	0.246	2	0.884
scale(prestim):verb:case:sag.	1.474	2	0.478
scale(prestim):verb:lat.:sag.	1.391	2	0.499
scale(prestim):case:lat.:sag.	0.702	1	0.402
verb:case:lat.:sag.	0.506	2	0.776
scale(prestim):verb:case:scale(epoch)	14.839	2	0.001
scale(prestim):verb:lat.:scale(epoch)	2.729	2	0.255
scale(prestim):case:lat.:scale(epoch)	4.570	1	0.033
verb:case:lat.:scale(epoch)	2.832	2	0.243
scale(prestim):verb:sag.:scale(epoch)	2.489	2	0.288
scale(prestim):case:sag.:scale(epoch)	0.029	1	0.865
verb:case:sag.:scale(epoch)	15.361	2	0.000
scale(prestim):lat.:sag.:scale(epoch)	0.010	1	0.919
verb:lat.:sag.:scale(epoch)	0.401	2	0.818
case:lat.:sag.:scale(epoch)	0.031	1	0.861
scale(prestim):verb:case:lat.:sag.	0.348	2	0.840
scale(prestim):verb:case:lat.:scale(epoch)	0.895	2	0.639
scale(prestim):verb:case:sag.:scale(epoch)	9.679	2	0.008
scale(prestim):verb:lat.:sag.:scale(epoch)	0.079	2	0.961
scale(prestim):case:lat.:sag.:scale(epoch)	2.172	1	0.141
verb:case:lat.:sag.:scale(epoch)	0.069	2	0.966
scale(prestim):verb:case:lat.:sag.:scale(epoch)	2.233	2	0.327

**Table 3:** Summary of effects in Late Positivity time window (Type II Wald Tests).

	**Chisq**	**Df**	**Pr(>Chisq)**
scale(prestim)	30281.850	1	0.000
verb	10.227	2	0.006
case	10.718	1	0.001
lat.	55.111	1	0.000
sag.	147.238	1	0.000
scale(epoch)	12.656	1	0.000
scale(prestim):verb	126.364	2	0.000
scale(prestim):case	0.189	1	0.664
verb:case	7.516	2	0.023
scale(prestim):lat.	26.950	1	0.000
verb:lat.	2.196	2	0.334
case:lat.	0.487	1	0.485
scale(prestim):sag.	112.018	1	0.000
verb:sag.	48.394	2	0.000
case:sag.	113.850	1	0.000
lat.:sag.	37.826	1	0.000
scale(prestim):scale(epoch)	11.877	1	0.001
verb:scale(epoch)	7.696	2	0.021
case:scale(epoch)	5.780	1	0.016
lat.:scale(epoch)	0.403	1	0.526
sag.:scale(epoch)	3.341	1	0.068
scale(prestim):verb:case	27.713	2	0.000
scale(prestim):verb:lat.	1.539	2	0.463
scale(prestim):case:lat.	0.414	1	0.520
verb:case:lat.	0.966	2	0.617
scale(prestim):verb:sag.	5.904	2	0.052
scale(prestim):case:sag.	1.541	1	0.215
verb:case:sag.	20.950	2	0.000
scale(prestim):lat.:sag.	0.138	1	0.710
verb:lat.:sag.	0.141	2	0.932
case:lat.:sag.	0.510	1	0.475
scale(prestim):verb:scale(epoch)	97.860	2	0.000
scale(prestim):case:scale(epoch)	0.120	1	0.729
verb:case:scale(epoch)	1.782	2	0.410
scale(prestim):lat.:scale(epoch)	0.002	1	0.964
verb:lat.:scale(epoch)	1.694	2	0.429
case:lat.:scale(epoch)	0.523	1	0.469
scale(prestim):sag.:scale(epoch)	4.024	1	0.045
verb:sag.:scale(epoch)	6.987	2	0.030
case:sag.:scale(epoch)	2.983	1	0.084
lat.:sag.:scale(epoch)	0.028	1	0.867
scale(prestim):verb:case:lat.	0.043	2	0.979
scale(prestim):verb:case:sag.	0.599	2	0.741
scale(prestim):verb:lat.:sag.	0.281	2	0.869
scale(prestim):case:lat.:sag.	0.020	1	0.887
verb:case:lat.:sag.	0.169	2	0.919
scale(prestim):verb:case:scale(epoch)	16.156	2	0.000
scale(prestim):verb:lat.:scale(epoch)	2.839	2	0.242
scale(prestim):case:lat.:scale(epoch)	4.166	1	0.041
verb:case:lat.:scale(epoch)	4.350	2	0.114
scale(prestim):verb:sag.:scale(epoch)	6.401	2	0.041
scale(prestim):case:sag.:scale(epoch)	0.164	1	0.686
verb:case:sag.:scale(epoch)	0.837	2	0.658
scale(prestim):lat.:sag.:scale(epoch)	0.651	1	0.420
verb:lat.:sag.:scale(epoch)	0.658	2	0.720
case:lat.:sag.:scale(epoch)	0.618	1	0.432
scale(prestim):verb:case:lat.:sag.	0.146	2	0.930
scale(prestim):verb:case:lat.:scale(epoch)	1.014	2	0.602
scale(prestim):verb:case:sag.:scale(epoch)	1.385	2	0.500
scale(prestim):verb:lat.:sag.:scale(epoch)	0.040	2	0.980
scale(prestim):case:lat.:sag.:scale(epoch)	2.724	1	0.099
verb:case:lat.:sag.:scale(epoch)	0.546	2	0.761
scale(prestim):verb:case:lat.:sag.:scale(epoch)	2.591	2	0.274

**Figure 6:**
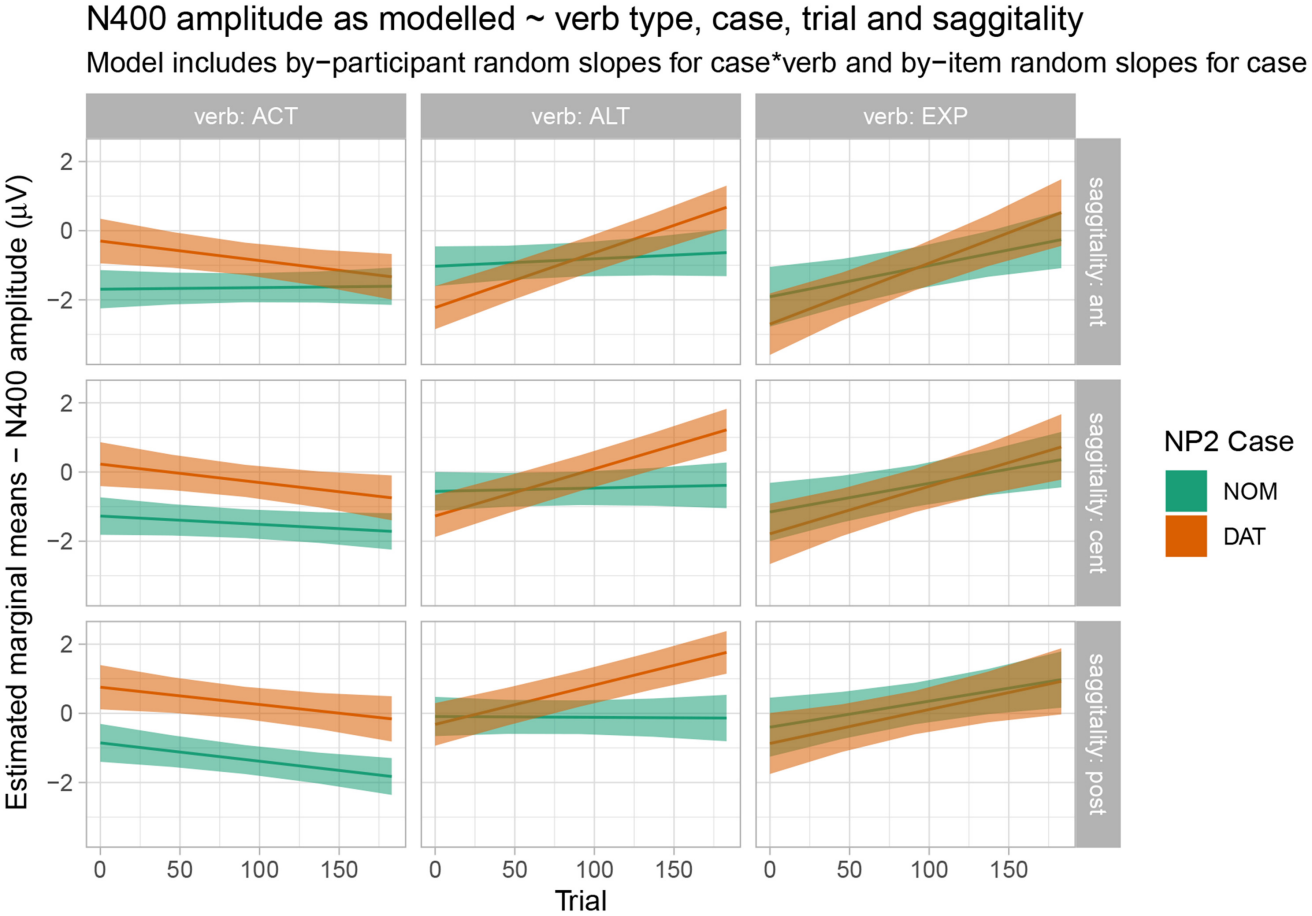
Estimated marginal means for the N400 time window by verb type, case, epoch, and saggitality. Shaded regions indicate 83% confidence intervals.

In the N400 time window, Wald tests revealed an interaction of verb x case x sagittality x epoch. This interaction is resolved and visualized in Figure 6, which shows estimated marginal means and 83% confidence intervals. As noted above for the behavioral data, non-overlap of 83% confidence intervals corresponds to a significant difference at the 5% level. It is apparent from Figure 6 that, for active verbs, dative-nominative orders show a negativity in comparison to nominative-dative orders over the course of the entire experiment. This effect is clearest in central and posterior regions. Alternating verbs, by contrast, do not show a clear pattern at the beginning of the experiment, but an N400 effect for dative-nominative vs. nominative-dative orders emerges over time and is clearly apparent in central and posterior regions by the end of the experiment. Experiencer verbs do not show any differential N400 effects for the two word orders at any point over the course of the experiment. Figures S7–S9, which serve to resolve the additional prestimulus interval x verb x case x sagittality x epoch interaction, show that this overall pattern is broadly consistent across a range of values of prestimulus amplitude from −5 to 5 μV^3^.

Please note that the relatively broad distribution of the N400 effects observed here (i.e., the fact that these effects weren't confined to centro-posterior sites but were also observable at more anterior channels) is consistent with the existing literature. A number of previous studies examining case-based processing mismatches have reported similarly broad N400 distributions (e.g., Frisch and Schlesewsky, 2001; Mueller et al., 2005).

**Figure 7:**
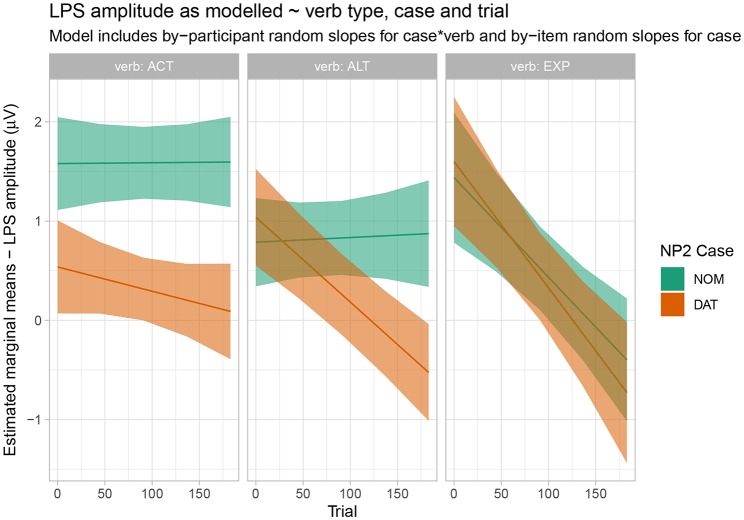
Estimated marginal means for the late positivity time window by verb type, case, and saggitality. Shaded regions indicate 83% confidence intervals.

For the late positivity time window, Wald tests showed an interaction of prestimulus amplitude x verb x case x epoch, which is resolved and visualized in Figure 7. Active verbs show a clear positivity for dative-nominative vs. nominative-dative sentences. For alternating verbs, a similar effect emerges over the course of the experiment. Finally, experiencer verbs show no indication of a late positivity effect for one word order as compared to the other.

Figures S10–S12 illustrate the verb x case x epoch interaction for different values of prestimulus amplitude. As for the N400, effects are consistent across a range of prestimulus values.

### 3.3. Acceptability-Contingent Analyses of ERPs to Dative Subject-Experiencer Verbs

For the dative subject-experiencer verbs, we conducted an additional analysis in order to examine whether the overall component pattern—i.e., the absence of N400 / late positivity effects differentiating between word orders—might be a reflection of the high variability of acceptability ratings for these verbs (cf. Figure 1). To this end, we fit a mixed model to the experiencer verb data in which we added single trial acceptability (acceptable:1, unacceptable:0) as an additional fixed factor. In view of the restriction to only one type of verb, the factor verb was no longer included in the model (both fixed and random effects). All other parameters remained as described above for the general ERP models and models of this type were fit for both the N400 and late positivity time windows.

In line with our hypotheses, we focus on interactions of acceptability and case and, for each statistical model, interpret the highest-order interaction involving both of these predictors.

**Figure 8:**
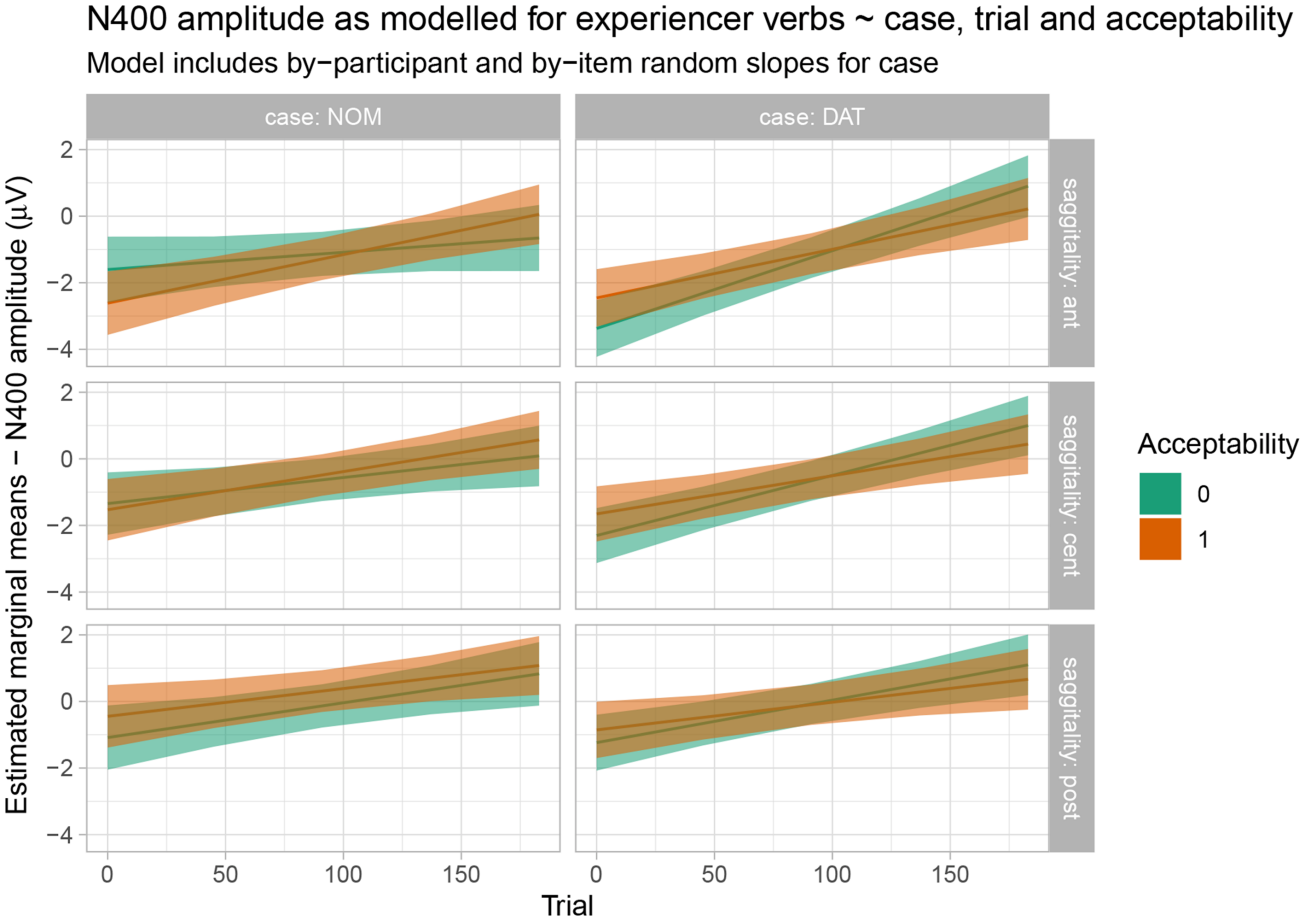
Estimated marginal means for the response-contingent analysis of the experiencer verb data in the N400 time window. Shaded regions indicate 83% confidence intervals.

In the N400 time window, Wald tests (cf. Table 4) showed an interaction of case x acceptability x sagittality x epoch, which is visualized and resolved in Figure 8. As is apparent from the figure, in spite of the interaction, there is no evidence for acceptability-based differences for either word order and this holds across the course of the experiment and for the different levels of sagittality.

**Table 4:** Summary of experiencer verb analysis including acceptability effects in N400 time window (Type II Wald Tests).

	**Chisq**	**Df**	**Pr(>Chisq)**
scale(prestim)	206.045	1	0.000
case	0.106	1	0.745
accept	2.781	1	0.095
lat.	69.315	1	0.000
sag.	293.656	1	0.000
scale(epoch)	9.880	1	0.002
scale(prestim):case	0.070	1	0.791
scale(prestim):accept	15.022	1	0.000
case:accept	0.701	1	0.402
scale(prestim):lat.	2.780	1	0.095
case:lat.	0.038	1	0.844
accept:lat.	0.276	1	0.599
scale(prestim):sag.	22.032	1	0.000
case:sag.	2.325	1	0.127
accept:sag.	0.851	1	0.356
lat.:sag.	0.701	1	0.402
scale(prestim):scale(epoch)	2.949	1	0.086
case:scale(epoch)	0.287	1	0.592
accept:scale(epoch)	4.888	1	0.027
lat.:scale(epoch)	0.146	1	0.703
sag.:scale(epoch)	15.625	1	0.000
scale(prestim):case:accept	34.062	1	0.000
scale(prestim):case:lat.	1.224	1	0.269
scale(prestim):accept:lat.	0.088	1	0.767
case:accept:lat.	1.484	1	0.223
scale(prestim):case:sag.	3.926	1	0.048
scale(prestim):accept:sag.	5.849	1	0.016
case:accept:sag.	6.477	1	0.011
scale(prestim):lat.:sag.	0.177	1	0.674
case:lat.:sag.	0.491	1	0.483
accept:lat.:sag.	0.954	1	0.329
scale(prestim):case:scale(epoch)	0.805	1	0.370
scale(prestim):accept:scale(epoch)	58.182	1	0.000
case:accept:scale(epoch)	8.719	1	0.003
scale(prestim):lat.:scale(epoch)	0.604	1	0.437
case:lat.:scale(epoch)	3.491	1	0.062
accept:lat.:scale(epoch)	0.143	1	0.705
scale(prestim):sag.:scale(epoch)	1.119	1	0.290
case:sag.:scale(epoch)	3.957	1	0.047
accept:sag.:scale(epoch)	0.427	1	0.514
lat.:sag.:scale(epoch)	0.002	1	0.963
scale(prestim):case:accept:lat.	0.753	1	0.386
scale(prestim):case:accept:sag.	0.239	1	0.625
scale(prestim):case:lat.:sag.	0.034	1	0.853
scale(prestim):accept:lat.:sag.	0.917	1	0.338
case:accept:lat.:sag.	0.635	1	0.426
scale(prestim):case:accept:scale(epoch)	5.886	1	0.015
scale(prestim):case:lat.:scale(epoch)	0.044	1	0.833
scale(prestim):accept:lat.:scale(epoch)	3.992	1	0.046
case:accept:lat.:scale(epoch)	0.013	1	0.909
scale(prestim):case:sag.:scale(epoch)	0.434	1	0.510
scale(prestim):accept:sag.:scale(epoch)	4.707	1	0.030
case:accept:sag.:scale(epoch)	5.816	1	0.016
scale(prestim):lat.:sag.:scale(epoch)	0.095	1	0.758
case:lat.:sag.:scale(epoch)	0.014	1	0.906
accept:lat.:sag.:scale(epoch)	0.200	1	0.655
scale(prestim):case:accept:lat.:sag.	0.001	1	0.977
scale(prestim):case:accept:lat.:scale(epoch)	1.219	1	0.270
scale(prestim):case:accept:sag.:scale(epoch)	0.256	1	0.613
scale(prestim):case:lat.:sag.:scale(epoch)	0.058	1	0.810
scale(prestim):accept:lat.:sag.:scale(epoch)	0.156	1	0.693
case:accept:lat.:sag.:scale(epoch)	0.003	1	0.957
scale(prestim):case:accept:lat.:sag.:scale(epoch)	0.000	1	0.997

**Figure 9:**
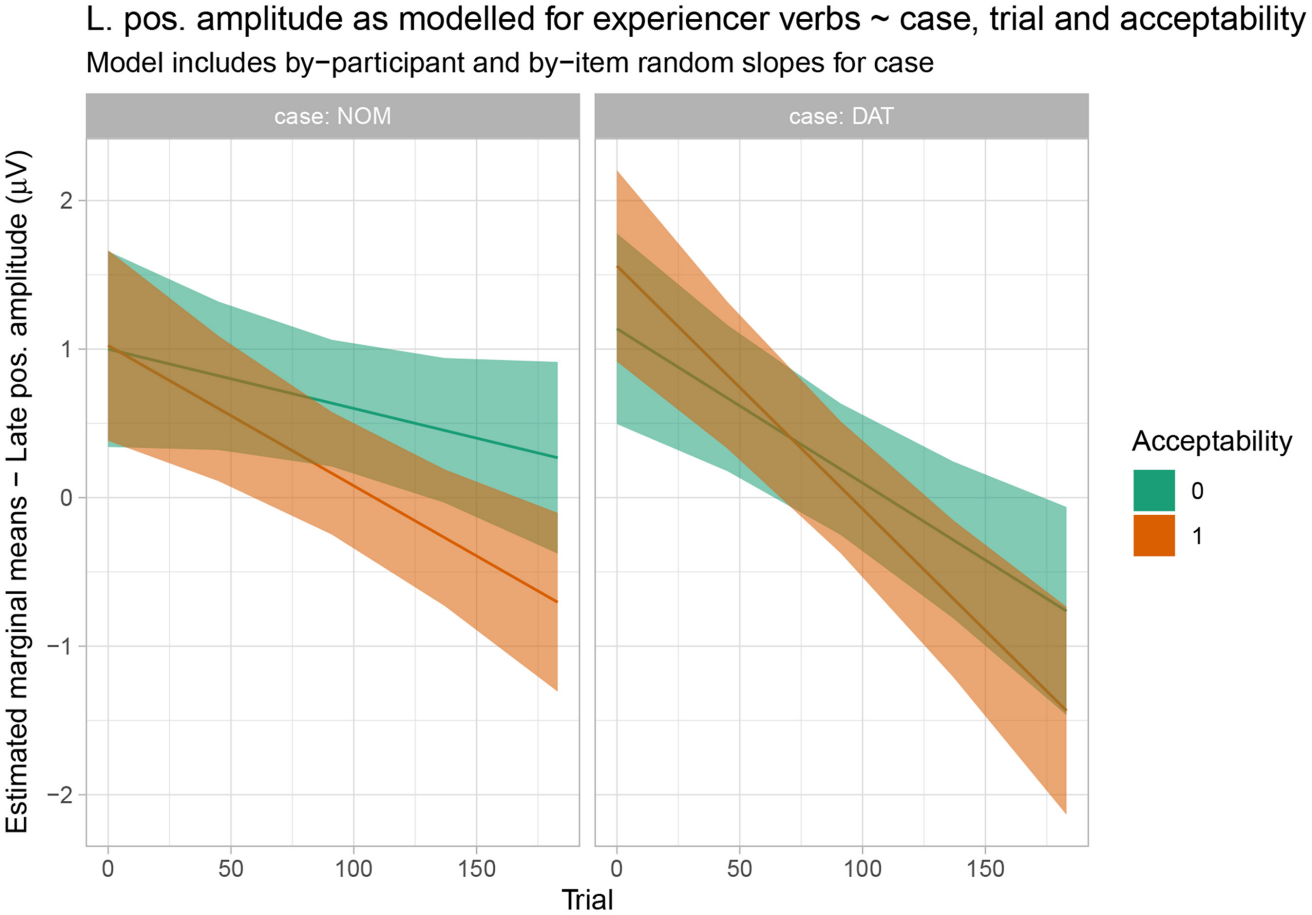
Estimated marginal means for the response-contingent analysis of the experiencer verb data in the late positivity time window. Shaded regions indicate 83% confidence intervals.

For the late positivity time window, we observed an interaction of case x acceptability x epoch x prestimulus amplitude (cf. Table 5). This interaction is visualized and resolved in Figure 9. Again, there is no evidence for acceptability-based differences and this pattern is broadly consistent across a range of prestimulus amplitudes (cf. Figures S13–S15).

**Table 5:** Summary of experiencer verb analysis including acceptability effects in late positivity time window (Type II Wald Tests).

	**Chisq**	**Df**	**Pr(>Chisq)**
scale(prestim)	8192.291	1	0.000
case	0.350	1	0.554
accept	13.170	1	0.000
lat.	16.012	1	0.000
sag.	8.666	1	0.003
scale(epoch)	15.275	1	0.000
scale(prestim):case	0.459	1	0.498
scale(prestim):accept	6.446	1	0.011
case:accept	8.356	1	0.004
scale(prestim):lat.	1.835	1	0.176
case:lat.	0.111	1	0.739
accept:lat.	0.075	1	0.784
scale(prestim):sag.	17.865	1	0.000
case:sag.	5.357	1	0.021
accept:sag.	0.615	1	0.433
lat.:sag.	10.965	1	0.001
scale(prestim):scale(epoch)	57.758	1	0.000
case:scale(epoch)	1.331	1	0.249
accept:scale(epoch)	24.118	1	0.000
lat.:scale(epoch)	0.266	1	0.606
sag.:scale(epoch)	2.820	1	0.093
scale(prestim):case:accept	91.025	1	0.000
scale(prestim):case:lat.	0.363	1	0.547
scale(prestim):accept:lat.	0.263	1	0.608
case:accept:lat.	0.275	1	0.600
scale(prestim):case:sag.	2.001	1	0.157
scale(prestim):accept:sag.	1.847	1	0.174
case:accept:sag.	0.009	1	0.925
scale(prestim):lat.:sag.	0.018	1	0.894
case:lat.:sag.	0.048	1	0.826
accept:lat.:sag.	1.170	1	0.279
scale(prestim):case:scale(epoch)	0.794	1	0.373
scale(prestim):accept:scale(epoch)	37.292	1	0.000
case:accept:scale(epoch)	0.157	1	0.692
scale(prestim):lat.:scale(epoch)	0.105	1	0.746
case:lat.:scale(epoch)	5.341	1	0.021
accept:lat.:scale(epoch)	2.350	1	0.125
scale(prestim):sag.:scale(epoch)	4.474	1	0.034
case:sag.:scale(epoch)	0.175	1	0.676
accept:sag.:scale(epoch)	2.593	1	0.107
lat.:sag.:scale(epoch)	0.146	1	0.702
scale(prestim):case:accept:lat.	1.059	1	0.303
scale(prestim):case:accept:sag.	2.148	1	0.143
scale(prestim):case:lat.:sag.	0.054	1	0.816
scale(prestim):accept:lat.:sag.	0.649	1	0.421
case:accept:lat.:sag.	0.114	1	0.735
scale(prestim):case:accept:scale(epoch)	4.018	1	0.045
scale(prestim):case:lat.:scale(epoch)	0.481	1	0.488
scale(prestim):accept:lat.:scale(epoch)	3.599	1	0.058
case:accept:lat.:scale(epoch)	2.389	1	0.122
scale(prestim):case:sag.:scale(epoch)	0.004	1	0.951
scale(prestim):accept:sag.:scale(epoch)	2.486	1	0.115
case:accept:sag.:scale(epoch)	1.164	1	0.281
scale(prestim):lat.:sag.:scale(epoch)	0.336	1	0.562
case:lat.:sag.:scale(epoch)	0.021	1	0.884
accept:lat.:sag.:scale(epoch)	0.288	1	0.591
scale(prestim):case:accept:lat.:sag.	0.000	1	0.983
scale(prestim):case:accept:lat.:scale(epoch)	0.002	1	0.966
scale(prestim):case:accept:sag.:scale(epoch)	0.018	1	0.892
scale(prestim):case:lat.:sag.:scale(epoch)	0.083	1	0.773
scale(prestim):accept:lat.:sag.:scale(epoch)	1.786	1	0.181
case:accept:lat.:sag.:scale(epoch)	0.649	1	0.420
scale(prestim):case:accept:lat.:sag.:scale(epoch)	0.919	1	0.338

In summary, there is no evidence that the ERP effects for the experiencer verbs vary on the basis of trial-by-trial changes in acceptability (full model summaries for the N400 and late positivity time windows are presented in Tables S7–S10).

## 4. Discussion

We have presented an ERP experiment on Icelandic, with which we aimed to examine whether transitional processes of language change may be observable in the neural correlates of language comprehension prior to the change manifesting itself in overt, language-related behavior. The rationale behind this research question was that processes of language change affecting word order tend to arise from the need to process information that is increasingly ambiguously marked. In other words, if case marking is perceived as increasingly ambiguous, this can lead to a reinterpretation that in turn results in a stricter constituent order. We hypothesized that this type of reinterpretation should manifest itself in ERP responses during online language comprehension. If present, it would also constitute a highly interesting phenomenon at the interface between brain and behavior—both at the level of individual speakers and in regard to the relation between neural processes, individual speaker behavior, and changes within communities of speakers.

We indeed observed a pattern of results that was highly compatible with our hypotheses, i.e., a pattern suggesting that the transition from one grammar to another manifests itself in processing patterns at the neural level even before becoming apparent in overt language behavior (in the case of our study: assessment of sentence acceptability). In the following, we first summarize our results and explain why we believe they support this position. We then go on to discuss how the two ERP components observed—the N400 and late positivity—map onto behavior, before considering the implications of our findings for theories of language processing and language change.

### 4.1. Summary: Language Processing Precedes Language Change

As noted above, we contend that our results are consistent with the hypothesis that changes in language processing can precede overt language change. We base this claim on the 2-fold pattern of acceptability ratings and ERP patterns observed in the present study. As we discuss in detail below, for each of the two verb types—alternating (ALT) and dative subject experiencer (EXP)—that we assume are undergoing a transition to the new target pattern (nominative subject, dative object), we observed a behavioral acceptability pattern that was “one step ahead” of what would be expected by the prescriptive grammar and an ERP pattern that was, in turn, one step ahead of the acceptability pattern.

Let us first consider the EXP verbs. Recall that, for these verbs, the prescriptive grammar requires dative subject and nominative object marking. From this perspective, they should thus be expected to show a pattern that is the mirror image of the one observed for active (ACT) verbs. However, while the behavioral ratings indeed show a higher acceptability for the dative-nominative (i.e., NP2 = nominative) as opposed to the nominative-dative (i.e., NP2 = dative) pattern for this verb class, the difference between the two patterns is not nearly as pronounced as the difference for nominative-dative vs. dative-nominative for ACT verbs (cf. Figure 2). In addition, EXP verbs also show highly variable judgement patterns across both participants and items (i.e., individual verbs; cf. Figure 1). This suggests that language change is already underway for this verb class, with both individual speakers and individual verbs differing with regard to how far the change has already advanced^4^. Crucially, the ERP patterns observed for the EXP verbs are indicative of an even further advanced degree of change in that the prescriptively ungrammatical order conforming to the target state of Grammar B (nominative-dative) did not differ neurophysiologically from the grammatical (Grammar A) order dative-nominative. As both of these structures constitute an optimal realization in one of the two grammars, neither shows increased real-time processing costs relative to the other. This speaks in favor of a growing influence of Grammar B on the language comprehension architecture, in which it apparently already coexists with Grammar A for these particular structures—at least during online processing. We interpret the absence of differential ERP effects for this verb class as indicating that case marking has become relatively uninformative for online interpretation. Hence, case marking patterns that are unexpected from the perspective of the current (prescriptive) Icelandic grammar—and even from the perspective of participants' own acceptability judgements—do not engender the typical ERP effects that are known to accompany these mismatches (N400, late positivity). The response-contingent analysis of the trial-by-trial ERP responses to experiencer verbs further supports this interpretation by demonstrating that the apparent absence of an effect cannot be explained by a trial-by-trial fluctuation of ERP responses depending on whether the construction was judged to be acceptable on a particular trial or not (i.e., it was not the case that sentences judged to be unacceptable engendered an N400-late positivity response irrespective of the case marking pattern). Our interpretation that case marking is no longer informative for online argument interpretation in these types of experiencer constructions in Icelandic is additionally corroborated by the observed pattern of comprehension accuracy, which was generally lower than that for the other two verb classes and did not differ depending on word order.

For the ALT verbs, the transition toward Grammar B is already much further advanced. Despite the possible grammatical dative-before-nominative realization (licensed by Grammar A), these verbs show a very similar and only slightly weaker neurophysiological response to that for the ACT verbs, in which the dative-before-nominative order is completely ruled out. Even though Grammar B obviously already dominates the processing of these structures, the weaker disadvantage for the dative-initial word order in comparison to the active verbs reflects the remaining remnants of Grammar A's influence, as does the higher degree of by-participant and by-item variability for ALT verbs (cf. Figures S3, S4). Strikingly, while the alternating verbs show no difference between word orders in the N400 at the beginning of the experiment, they converge on the pattern shown by the active verbs (increased N400 amplitude for dative-nominative vs. nominative-dative orders) by the end of the experimental session (see Figure 4). We take this to reflect the higher degree of uncertainty surrounding the dominant or preferred structure with these verbs in comparison to active verbs. Supporting this notion, there is a high degree of judgement variability for the dative-nominative pattern with alternating verbs (Figure 1)—paralleling that for the experiencer verbs. The nominative-dative order, by contrast, is consistently judged as acceptable, thus patterning with the results for the active verbs. Comprehension accuracy mirrors these results in that participants were highly accurate in responding to the comprehension questions for alternating verbs with nominative-dative orders, but considerably less accurate in the case of dative-nominative orders.

Finally, the active verbs showed a highly consistent pattern across all the measures employed here, as was expected given that they already conform to the requirements of the target grammar (B). These verbs showed a clear N400—late positivity pattern for dative-nominative vs. nominative-dative orders, which was apparent across the entire experiment. Nominative-dative orders were consistently judged to be acceptable across participants and items, while dative-nominative orders were consistently rejected. Intriguingly, the results of the comprehension task revealed that sentences with active verbs were comprehended highly accurately independently of the word order. This was the case in spite of the low acceptability of the dative-nominative order. We interpret this pattern as being indicative of low interpretative value of case marking in these structures: all that matters for comprehension is which argument occupies the subject position. This is reminiscent of how language comprehension operates in modern English, in which word order always dominates morphological marking as an interpretative cue.

### 4.2. The Relation Between the N400 and Late Positivity Components and Behavior

Having discussed our interpretation of the overall pattern of results, we now turn to a more mechanistic account of what we consider the N400 and late positivity components to reflect in the current data.

#### 4.2.1. N400

We have recently proposed that N400 effects reflect precision-weighted prediction errors (Bornkessel-Schlesewsky and Schlesewsky, 2019) in the sense of a predictive-coding account of brain function (cf. Friston, 2005, 2010). In brief, predictive coding assumes that the brain actively constructs explanations for its sensory input and that this involves maintaining an internal generative (predictive) model of the world around us. The brain is thus constantly engaged in generating predictions for upcoming sensory input and in matching these to the input actually encountered. Prediction errors (i.e., mismatches between prediction and input) can lead to internal model updating. Crucially, predictions differ in regard to their precision, which is defined as the inverse of variance and thus essentially reflects the degree of (un)certainty (Feldman and Friston, 2010). Prediction precision has been shown to modulate mismatch negativity (MMN) effects (Todd et al., 2014) and, as posited in Bornkessel-Schlesewsky and Schlesewsky (2019), there is evidence to suggest that the same holds for N400 effects in language. From this perspective, we would expect to observe more pronounced N400 effects for higher precision predictions. This approach constitutes a promising conceptual framework for interpreting the N400 effects in the current experiment (for a comparison to other current interpretations of the N400, see Bornkessel-Schlesewsky and Schlesewsky, 2019).

In sentences with active verbs, the language comprehension system is able to generate a high-precision prediction for a post-verbal dative argument. When this prediction is not borne out, the resulting prediction error is reflected in an N400 effect. The prediction (and precision of the prediction) is highly stable, thus leading to comparable N400 effects across the course of the experiment for active verbs.

For alternating verbs, the situation is more complex. While the nominative-dative order is highly acceptable across the board, it has a competitor in the dative-nominative order—with the degree of competition varying across participants and items. Accordingly, there is a lower precision prediction for the case marking of the post-verbal NP and no N400 difference at the beginning of experiment. Across the course of the experiment, however, the precision of the prediction for nominative-dative appears to strengthen, and an N400 effect emerges. We speculate that this by-trial change may have been precipitated by the presence of a high number of active verbs in the experiment. (But note that there was no comparable emergence of an N400 effect for the experiencer verbs, thus suggesting that alternating verbs were more strongly susceptible to such an influence). Yet whatever the explanation for the emergence of an N400 effect for dative-nominative vs. nominative-dative orders, this pattern attests to a less stable pattern than that for the active verbs, as also seen in the behavioral data. For the alternating verbs, uncertainty arising from the variability in the dative-nominative order is key to the overall pattern of results.

The experiencer verbs show a high behavioral uncertainty for both word orders. Thus, predictions in online processing are of a very low precision and this manifests itself in the absence of reliable N400 effects in either direction^5^.

#### 4.2.2. Late Positivity

The late positivity effects in the current experiment showed a similar pattern to those observed in the N400: active verbs showed a positivity for dative-nominative vs. nominative-dative orders across the entire experiment; for alternating verbs, a similar effect emerged over the course of the experiment; experiencer verbs showed no differential late positivity effects. Overall, the late positivity appears to reflect the dominant acceptability pattern for each verb class: a clear preference for nominative-dative for active verbs; a similar, but weaker preference for alternating verbs; and high variability for experiencer verbs. In spite of the generally similar patterns for the N400 and late positivity, we expected that the late positivity effects observed should be tied more strongly to the overall evaluation of the structures in question than to their incremental comprehension (and the prediction-based effects involved therein). We derive this assumption from the proposal that late positivity effects in language should be viewed as members of the P300 family (e.g., Coulson et al., 1998; Sassenhagen et al., 2014) and that they are therefore connected more closely to the motivational salience of a stimulus and how this translates to behavior (for discussion in comparison to the N400, see Bornkessel-Schlesewsky and Schlesewsky, 2019).

In order to test this assumption further, we computed two generalized linear mixed models, in which we examined the extent to which single-trial N400 and late positivity amplitudes can predict single-trial acceptability ratings. We included (z-transformed) mean amplitude for the respective time window, laterality and sagittality in the model as fixed effects, with random intercepts grouped by participant and item. Both the N400 and the late positivity model fits were improved by additionally adding verb type as a predictor [likelihood ratio test for N400 model: χ^2^(16) = 86.88, *p* < 0.001; late positivity model: χ^2^(16) = 262.55, *p* < 0.001]. While both N400 and late positivity amplitudes predicted acceptability on a single trial basis [N400 amplitude x verb type: χ^2^(2) = 59.76, *p* < 0.001; LPS amplitude x verb type: χ^2^(2) = 218.02, *p* < 0.001], the late positivity model showed an overall better fit to the data (AIC for N400 model including verb type: 114407; AIC for late positivity model including verb type: 113320). In addition, as shown in Figures S16, S17, late positivity amplitudes showed a stronger relationship with acceptability than N400 amplitudes. Interestingly, in both times windows, EEG amplitudes were more strongly predictive of acceptability for active and alternating than for experiencer verbs, thus further supporting our argument of highly variable EEG responses for experiencer verbs that are not correlated with acceptability.

In summary, single-trial late positivity amplitudes were more predictive of behavior (acceptability) than N400 amplitudes, as expected. Thus, in spite of the fact that the late positivity effects observed here showed larger amplitudes than the N400 effects, we suggest that the N400 effects will be more predictive of language change due to their higher sensitivity to the demands of online comprehension and stronger independence from behavior. Whether this assumption is indeed correct, however, cannot be determined on the basis of the present findings, since our study does not include any longitudinal or diachronic data. It should therefore be viewed as a testable hypothesis for future research based on the current results, rather than as a conclusion from the current study.

### 4.3. Implications for The Relation Between Language Processing and Language Change

These findings provide initial converging evidence for an intriguing picture of the dynamics of language change. In particular, they suggest that we can identify three successively less conservative levels of language behavior: (a) the prescriptive grammar and conscious behavior adhering to its rules; (b) the intuitions of native speakers under time pressure—and thereby under similar circumstances as in real life communication; and (c) the underlying source of all of these behavioral responses: the human brain. These three dimensions are ordered hierarchically with respect to one another, such that each is “one step ahead” of the previous stage^6^. While it is well-known that changes in prescriptive grammar result from an adaptation to transitions that have already been established in everyday language use, our findings suggest that the neural processing architecture in turn paves the way for these changes in overt language-based behavior. Brain responses—which, as discussed in the introduction, can be viewed as reflecting the reintepretation processes that foreshadow at least certain processes of language change—can therefore be used as early indicators for transitions that will subsequently emerge in first the informal and later the formal (normative) uses of a particular language. Depending on the particular neurophysiological patterns observed, concrete predictions for the direction of language change can be formulated.

Regarding Icelandic, our data suggest that the alternating verbs will come to be associated with a fixed nominative-initial word order, thereby completing a change that is already relatively far advanced. More interestingly, the dative subject-experiencer verbs are predicted to first turn into alternating verbs in both surface behavior and prescriptive grammar, before following the current alternating verbs on their path toward the fixed nominative-first active constructions. As a consequence, dative subjects in Icelandic will become first an endangered and subsequently an extinct species. This will likely be the starting point for a complete deconstruction of the morphological system.

As noted above, we suggest that N400 effects may be particularly promising early indicators of the initial stages of such a process, namely reinterpretation during language processing. The proposal that N400 effects reflect precision-weighted prediction errors provides a neurobiological grounding for this claim: as an information source becomes more ambiguous, it becomes less reliable for formulating predictions and any predictions generated during online comprehension are thus of lower precision. Reduced N400 effects to structures that are incompatible with the current prescriptive grammar could thus provide us with an early “snapshot of the brain in transition” and hence the capacity to predict the directions that languages will take in their future development.

## Data Availability Statement

Datasets are in a publicly accessible repository: The datasets generated for this study and the analysis code can be found in an Open Science Framework repository, https://osf.io/zp6yv/.

## Ethics Statement

The present study was performed in accordance with the ethical standards laid down in the Declaration of Helsinki. Participants gave written informed consent before the beginning of the experiment and were informed that they could discontinue the study at any time should they wish to do so. The experimental protocols were approved by the ethics committee of the Max Planck Institute for Human Cognitive and Brain Sciences, Leipzig, Germany.

## Author's Note

We are indebted to Jörgen Pind for the opportunity to acquire data at the Department of Psychology of the University of Iceland, Reykjavik. We would further like to thank Thórhallur Eythórsson for helpful discussions and Ella Björt Teague and Sigrúnsif Jóelsdóttir for assistance in data acquisition. This manuscript was written in RMarkdown using the R package *papaja* (Aust and Barth, 2018).

## Author Contributions

IB-S, MS, and DR designed the research. DR conducted the research. IB-S and DR analyzed the data. IB-S, MS, RM, and DR wrote the paper.

### Conflict of Interest

The authors declare that the research was conducted in the absence of any commercial or financial relationships that could be construed as a potential conflict of interest.

## Footnotes

^1^Note that we do not consider sociolinguistic perspectives on language change here, as they are outside the scope of what we aim to examine with this paper. Our study investigates whether shifts in processing (i.e., comprehension) strategies in individuals precede overt language change, i.e., if speakers change their perception before the propagation of any change that would require a sociolinguistic perspective, and if this could possibly be a trigger for language change.

^2^From the perspective of traditional grammar, “non-nominative subject” may appear to be a contradiction in terms because nominative marking is one of the classic defining properties of subjecthood. However, cross-linguistic research has revealed the existence of non-nominative subjects in a number of languages in all parts of the world (e.g., Hindi, Russian, Japanese) (Bhaskararao and Subbarao, 2004). While these arguments do not bear nominative case and often show no (or only reduced) agreement with the verb, they display a number of other subject properties e.g., with respect to control, reflexivization, deletion under conditions of coreference.

^3^As Alday (2019) notes in regard to interpreting interactions with prestimulus amplitudes: “As elsewhere in statistics, we can include additional covariates as controls without further interpreting those covariates. In other words, we can safely ignore the terms related to baseline correction, but we cannot omit them from the model.” (p. 9) We include the supplementary figures for completeness' sake and to explore whether the presence of different verb classes prior to our critical NP2 position may have had an effect. However, this does not appear to have been the case.

^4^Note, however, that since we did not collect any production data from our participants, we do not know whether an acceptance of the nominative-dative pattern for EXP verbs also correlates with their use of this pattern in language production. It is therefore possible that, while at least some participants find this pattern highly acceptable, they do not yet produce it themselves.

^5^We suggest that the different patterns for the experiencer vs. alternating verbs may reflect the fact that only the experiencer verbs are subject to two competing patterns: nominative-dative, which reflects the canonical subject-object pattern in terms of grammatical relations, and dative-nominative, which reflects the semantic role hierarchy Experiencer > Stimulus. In other words, the association between dative case marking and the Experiencer role serves to bolster the dative-nominative pattern for the experiencer verbs—this is likely also the mechanism underlying dative substitution for experiencer verbs with accusative subjects, cf. example 1. Thus, while the experiencer verbs are subject to a high level of uncertainty due to two competing case marking patterns based on grammatical relations and semantic roles, respectively, the dative-nominative pattern for alternating verbs is not supported by semantic information and thus more susceptible to change over the course of the experiment.

^6^Note that this assumption is further supported by the phenomenon of “case sickness” in Icelandic (cf. Eythórsson, 2000). Case sickness refers, for example, to dative case marking being used to mark the subjects of accusative subject verbs, a tendency that is common in Icelandic but frowned upon by prescriptive grammars. The motivation for this change is likely that dative is commonly used to mark actor-type arguments, while accusative is not. Hence, language production is one step ahead of the prescriptive grammar. At the same time, our results suggest that the change is already further advanced in language comprehension in that position is weighted more strongly than case for interpretation, possibly due to the reduction of the number of cases permissible in particular structural positions.
