# Supplementary material for: Language Processing as a Precursor to Language Change: Evidence From Icelandic
Source: Front Psychol. 2020 Jan 17;10:3013. doi: 10.3389/fpsyg.2019.03013 (PMC6978737; doi:10.3389/fpsyg.2019.03013)
Supplement: Supplementary file 1 [file Data_Sheet_1.pdf]

Supplementary Tables

Language processing as a precursor to language change: evidence from Icelandic

January 2, 2020

Table S1: Summary of model produced by the call `glmer(formula = f1 ~ verb * case + (1 | subj) + (1 | item), data = behav.s, family = "binomial")`
Generalized linear mixed model fit by maximum likelihood (Laplace Approximation)

AIC	BIC	logLik	deviance	df.resid
2935	2986	-1460	2919	4172

Scaled residuals:

Min	1Q	Median	3Q	Max
-10.38	-0.31	0.13	0.3	5.21

Random effects:

Groups	Term	Std.Dev.
item	(Intercept)	0.60793
subj	(Intercept)	0.55880

Number of obs: 4180, groups: item, 90; subj, 23.

Fixed effects:

	Estimate	Std. Error	z value	Pr(>|z|)	
(Intercept)	0.62	0.15	4.3	2.1e−05	***
verb[ALT]	0.1	0.13	0.83	0.41	
verb[EXP]	0.021	0.13	0.16	0.87	
case[NOM]	−1.4	0.056	−24	1e−127	***
verb[ALT]:case[NOM]	−0.31	0.075	−4.1	4.3e−05	***
verb[EXP]:case[NOM]	2.1	0.075	29	1.2e−178	***

Table S2: Summary of model produced by the call `glmer(formula = f2 ~ verb * case + (1 | subj) + (1 | item), data = behav.s, family = "binomial")`
Generalized linear mixed model fit by maximum likelihood (Laplace Approximation)

AIC	BIC	logLik	deviance	df.resid
3629	3680	-1807	3613	4139

Scaled residuals:

Min	1Q	Median	3Q	Max
-7.68	0.12	0.3	0.46	2.97

Random effects:

Groups	Term	Std.Dev.
item	(Intercept)	0.7073
subj	(Intercept)	1.0805

Number of obs: 4147, groups: item, 90; subj, 23.

Fixed effects:

	Estimate	Std. Error	z value	Pr(>|z|)	
(Intercept)	1.5	0.25	6.1	1.1e−09	***
verb[ALT]	0.4	0.14	2.9	0.0036	**
verb[EXP]	−0.93	0.14	−6.7	1.9e−11	***
case[NOM]	−0.18	0.045	−4.1	4.1e−05	***
verb[ALT]:case[NOM]	−0.38	0.069	−5.6	2.5e−08	***
verb[EXP]:case[NOM]	0.25	0.063	3.9	8e−05	***

Table S3: Summary of residuals and random effects for model produced by the call `lmer(formula = mean ~ scale(prestim) * verb * case * lat. * sag. * scale(epoch) + (1 + verb * case | subj) + (1 + case | item), data = filter(np2, wname == "n400"), REML = FALSE, control = lmerControl(optimizer = "bobyqa", calc.derivs = TRUE))`

Linear mixed model fit by maximum likelihood

AIC	BIC	logLik	deviance	df.resid
602205	603359	-300981	601963	102954

Scaled residuals:

Min	1Q	Median	3Q	Max
-5.82	-0.62	0.02	0.63	5.51

Random effects:

Groups	Term	Std.Dev.	Corr				
item	(Intercept)	0.94028					
	case[NOM]	0.73586	-0.288				
subj	(Intercept)	1.19192					
	verb[ALT]	0.57261	-0.493				
	verb[EXP]	0.52448	0.503	-0.909			
	case[NOM]	0.39871	0.116	-0.223	0.172		
	verb[ALT]:case[NOM]	0.43785	0.096	0.120	0.000	0.157	
	verb[EXP]:case[NOM]	0.51387	0.097	-0.045	-0.049	0.222	-0.723
Residual		4.46267					

Number of obs: 103075, groups: item, 90; subj, 23.

Table S4: Summary of fixed effects for model produced by the call `lmer(formula = mean ~ scale(prestim) * verb * case * lat. * sag. * scale(epoch) + (1 + verb * case | subj) + (1 + case | item), data = filter(np2, wname == "n400"), REML = FALSE, control = lmerControl(optimizer = "bobyqa", calc.derivs = TRUE))`.

Beginning of Table S4 (fixed effects N400)			
	Estimate	Std. Error	t value
(Intercept)	−0.55	0.27	−2
scale(prestim)	−0.67	0.016	−41
verb[ALT]	0.28	0.2	1.4
verb[EXP]	0.051	0.2	0.26
case[NOM]	−0.25	0.12	−2.1
lat.	0.39	0.026	15
sag.	−0.71	0.027	−27
scale(epoch)	0.25	0.082	3
scale(prestim):verb[ALT]	−0.13	0.024	−5.3
scale(prestim):verb[EXP]	0.23	0.025	9.4
scale(prestim):case[NOM]	0.033	0.016	2
verb[ALT]:case[NOM]	0.043	0.16	0.28
verb[EXP]:case[NOM]	0.33	0.17	1.9
scale(prestim):lat.	−0.13	0.031	−4.1
verb[ALT]:lat.	0.0051	0.038	0.13
verb[EXP]:lat.	0.032	0.04	0.8
case[NOM]:lat.	0.052	0.026	2
scale(prestim):sag.	0.24	0.028	8.6
verb[ALT]:sag.	−0.059	0.039	−1.5
verb[EXP]:sag.	−0.16	0.041	−3.8
case[NOM]:sag.	0.15	0.027	5.7
lat.:sag.	−0.08	0.053	−1.5
scale(prestim):scale(epoch)	0.042	0.017	2.5
verb[ALT]:scale(epoch)	0.13	0.1	1.3
verb[EXP]:scale(epoch)	0.32	0.14	2.4
case[NOM]:scale(epoch)	−0.13	0.078	−1.7
lat.:scale(epoch)	0.038	0.026	1.5
sag.:scale(epoch)	0.13	0.027	4.8
scale(prestim):verb[ALT]:case[NOM]	0.05	0.024	2.1
scale(prestim):verb[EXP]:case[NOM]	−0.048	0.025	−1.9
scale(prestim):verb[ALT]:lat.	−0.014	0.045	−0.32
scale(prestim):verb[EXP]:lat.	0.03	0.047	0.65
scale(prestim):case[NOM]:lat.	−0.068	0.031	−2.2
verb[ALT]:case[NOM]:lat.	0.016	0.038	0.42
verb[EXP]:case[NOM]:lat.	−0.042	0.04	−1
scale(prestim):verb[ALT]:sag.	−0.012	0.041	−0.29

Continuation of Table S4 (fixed effects N400)			
	Estimate	Std. Error	t value
scale(prestim):verb[EXP]:sag.	−0.038	0.042	−0.89
scale(prestim):case[NOM]:sag.	−0.12	0.028	−4.2
verb[ALT]:case[NOM]:sag.	0.12	0.039	3
verb[EXP]:case[NOM]:sag.	−0.24	0.041	−5.8
scale(prestim):lat.:sag.	0.051	0.06	0.85
verb[ALT]:lat.:sag.	−0.013	0.077	−0.17
verb[EXP]:lat.:sag.	0.0095	0.081	0.12
case[NOM]:lat.:sag.	0.0053	0.053	0.1
scale(prestim):verb[ALT]:scale(epoch)	0.058	0.023	2.6
scale(prestim):verb[EXP]:scale(epoch)	−0.12	0.027	−4.7
scale(prestim):case[NOM]:scale(epoch)	−0.053	0.017	−3.2
verb[ALT]:case[NOM]:scale(epoch)	−0.2	0.097	−2.1
verb[EXP]:case[NOM]:scale(epoch)	−0.0077	0.13	−0.061
scale(prestim):lat.:scale(epoch)	0.0099	0.032	0.32
verb[ALT]:lat.:scale(epoch)	−0.0021	0.036	−0.057
verb[EXP]:lat.:scale(epoch)	−0.0039	0.042	−0.093
case[NOM]:lat.:scale(epoch)	−0.035	0.026	−1.3
scale(prestim):sag.:scale(epoch)	0.06	0.028	2.1
verb[ALT]:sag.:scale(epoch)	−0.0057	0.037	−0.15
verb[EXP]:sag.:scale(epoch)	0.04	0.043	0.95
case[NOM]:sag.:scale(epoch)	−0.012	0.027	−0.45
lat.:sag.:scale(epoch)	−0.017	0.053	−0.32
scale(prestim):verb[ALT]:case[NOM]:lat.	0.024	0.045	0.53
scale(prestim):verb[EXP]:case[NOM]:lat.	−0.014	0.047	−0.29
scale(prestim):verb[ALT]:case[NOM]:sag.	−0.0042	0.041	−0.1
scale(prestim):verb[EXP]:case[NOM]:sag.	−0.035	0.042	−0.82
scale(prestim):verb[ALT]:lat.:sag.	0.11	0.089	1.3
scale(prestim):verb[EXP]:lat.:sag.	−0.085	0.092	−0.92
scale(prestim):case[NOM]:lat.:sag.	0.056	0.06	0.93
verb[ALT]:case[NOM]:lat.:sag.	−0.001	0.077	−0.014
verb[EXP]:case[NOM]:lat.:sag.	0.049	0.081	0.6
scale(prestim):verb[ALT]:case[NOM]:scale(epoch)	0.042	0.023	1.9
scale(prestim):verb[EXP]:case[NOM]:scale(epoch)	−0.1	0.027	−3.9
scale(prestim):verb[ALT]:lat.:scale(epoch)	0.067	0.043	1.6
scale(prestim):verb[EXP]:lat.:scale(epoch)	−0.04	0.051	−0.79
scale(prestim):case[NOM]:lat.:scale(epoch)	0.066	0.032	2.1
verb[ALT]:case[NOM]:lat.:scale(epoch)	0.035	0.036	0.98
verb[EXP]:case[NOM]:lat.:scale(epoch)	−0.071	0.042	−1.7
scale(prestim):verb[ALT]:sag.:scale(epoch)	0.046	0.039	1.2
scale(prestim):verb[EXP]:sag.:scale(epoch)	−0.084	0.046	−1.8
scale(prestim):case[NOM]:sag.:scale(epoch)	−0.035	0.028	−1.2
verb[ALT]:case[NOM]:sag.:scale(epoch)	−0.026	0.037	−0.69
verb[EXP]:case[NOM]:sag.:scale(epoch)	−0.1	0.043	−2.4
scale(prestim):lat.:sag.:scale(epoch)	0.016	0.062	0.26

Continuation of Table S4 (fixed effects N400)			
	Estimate	Std. Error	t value
verb[ALT]:lat.:sag.:scale(epoch)	0.042	0.073	0.58
verb[EXP]:lat.:sag.:scale(epoch)	−0.0078	0.084	−0.094
case[NOM]:lat.:sag.:scale(epoch)	0.0067	0.053	0.13
scale(prestim):verb[ALT]:case[NOM]:lat.:sag.	0.054	0.089	0.61
scale(prestim):verb[EXP]:case[NOM]:lat.:sag.	−0.036	0.092	−0.39
scale(prestim):verb[ALT]:case[NOM]:lat.:scale(epoch)	0.034	0.043	0.78
scale(prestim):verb[EXP]:case[NOM]:lat.:scale(epoch)	−0.0025	0.051	−0.05
scale(prestim):verb[ALT]:case[NOM]:sag.:scale(epoch)	−0.041	0.039	−1.1
scale(prestim):verb[EXP]:case[NOM]:sag.:scale(epoch)	−0.071	0.046	−1.6
scale(prestim):verb[ALT]:lat.:sag.:scale(epoch)	−0.034	0.085	−0.4
scale(prestim):verb[EXP]:lat.:sag.:scale(epoch)	0.028	0.099	0.28
scale(prestim):case[NOM]:lat.:sag.:scale(epoch)	−0.056	0.062	−0.9
verb[ALT]:case[NOM]:lat.:sag.:scale(epoch)	−0.011	0.073	−0.14
verb[EXP]:case[NOM]:lat.:sag.:scale(epoch)	−0.0084	0.084	−0.1
scale(prestim):verb[ALT]:case[NOM]:lat.:sag.:scale(epoch)	0.069	0.085	0.82
scale(prestim):verb[EXP]:case[NOM]:lat.:sag.:scale(epoch)	0.04	0.099	0.4
End of Table S4			

Table S5: Summary of residuals and random effects for model produced by the call `lmer(formula = mean ~ scale(prestim) * verb * case * lat. * sag. * scale(epoch) + (1 + verb * case | subj) + (1 + case | item), data = filter(np2, wname == "lps"), REML = FALSE, control = lmerControl(optimizer = "bobyqa", calc.derivs = TRUE))`

Linear mixed model fit by maximum likelihood

AIC	BIC	logLik	deviance	df.resid
574896	576051	-287327	574654	102954

Scaled residuals:

Min	1Q	Median	3Q	Max
-5.17	-0.64	-0.01	0.61	4.63

Random effects:

Groups	Term	Std.Dev.	Corr				
item	(Intercept)	0.64164					
	case[NOM]	0.66977	-0.193				
subj	(Intercept)	0.76280					
	verb[ALT]	0.38073	-0.307				
	verb[EXP]	0.30058	0.473	-0.602			
	case[NOM]	0.39712	0.266	-0.195	-0.016		
	verb[ALT]:case[NOM]	0.35260	0.036	-0.498	0.040	0.249	
	verb[EXP]:case[NOM]	0.54920	-0.047	0.419	-0.070	-0.281	-0.759
Residual		3.90973					

Number of obs: 103075, groups: item, 90; subj, 23.

Table S6: Summary of fixed effects for model produced by the call `lmer(formula = mean ~ scale(prestim) * verb * case * lat. * sag. * scale(epoch) + (1 + verb * case | subj) + (1 + case | item), data = filter(np2, wname == "lps"), REML = FALSE, control = lmerControl(optimizer = "bobyqa", calc.derivs = TRUE))`.

Beginning of Table S6 (fixed effects LPS)			
	Estimate	Std. Error	t value
(Intercept)	0.67	0.18	3.8
scale(prestim)	−2.3	0.014	−1.6e+02
verb[ALT]	−0.12	0.14	−0.87
verb[EXP]	−0.17	0.13	−1.3
case[NOM]	0.32	0.11	2.8
lat.	−0.15	0.023	−6.3
sag.	−0.21	0.024	−9.1
scale(epoch)	−0.29	0.065	−4.4
scale(prestim):verb[ALT]	0.18	0.021	8.8
scale(prestim):verb[EXP]	−0.23	0.022	−11
scale(prestim):case[NOM]	−0.02	0.014	−1.4
verb[ALT]:case[NOM]	−0.039	0.14	−0.29
verb[EXP]:case[NOM]	−0.28	0.16	−1.7
scale(prestim):lat.	0.12	0.027	4.5
verb[ALT]:lat.	0.045	0.033	1.3
verb[EXP]:lat.	−0.023	0.035	−0.65
case[NOM]:lat.	0.0031	0.023	0.14
scale(prestim):sag.	−0.21	0.024	−8.6
verb[ALT]:sag.	0.11	0.034	3.1
verb[EXP]:sag.	0.095	0.036	2.6
case[NOM]:sag.	−0.19	0.024	−8.3
lat.:sag.	0.26	0.046	5.6
scale(prestim):scale(epoch)	0.011	0.014	0.76
verb[ALT]:scale(epoch)	0.077	0.082	0.94
verb[EXP]:scale(epoch)	−0.31	0.11	−2.9
case[NOM]:scale(epoch)	0.12	0.066	1.9
lat.:scale(epoch)	0.022	0.023	0.95
sag.:scale(epoch)	−0.056	0.024	−2.4
scale(prestim):verb[ALT]:case[NOM]	−0.097	0.021	−4.7
scale(prestim):verb[EXP]:case[NOM]	0.035	0.022	1.6
scale(prestim):verb[ALT]:lat.	0.034	0.04	0.87
scale(prestim):verb[EXP]:lat.	−0.048	0.041	−1.2
scale(prestim):case[NOM]:lat.	0.015	0.027	0.56
verb[ALT]:case[NOM]:lat.	−0.0064	0.033	−0.19
verb[EXP]:case[NOM]:lat.	−0.024	0.035	−0.67
scale(prestim):verb[ALT]:sag.	0.017	0.036	0.46

Continuation of Table S6 (fixed effects LPS)			
	Estimate	Std. Error	t value
scale(prestim):verb[EXP]:sag.	0.053	0.037	1.4
scale(prestim):case[NOM]:sag.	0.035	0.024	1.4
verb[ALT]:case[NOM]:sag.	0.042	0.034	1.2
verb[EXP]:case[NOM]:sag.	0.09	0.036	2.5
scale(prestim):lat.:sag.	−0.031	0.053	−0.58
verb[ALT]:lat.:sag.	−0.017	0.067	−0.26
verb[EXP]:lat.:sag.	0.021	0.071	0.29
case[NOM]:lat.:sag.	0.027	0.046	0.57
scale(prestim):verb[ALT]:scale(epoch)	−0.13	0.02	−6.7
scale(prestim):verb[EXP]:scale(epoch)	0.24	0.023	10
scale(prestim):case[NOM]:scale(epoch)	0.029	0.014	2
verb[ALT]:case[NOM]:scale(epoch)	0.11	0.083	1.4
verb[EXP]:case[NOM]:scale(epoch)	−0.057	0.11	−0.52
scale(prestim):lat.:scale(epoch)	−0.0095	0.028	−0.34
verb[ALT]:lat.:scale(epoch)	0.026	0.032	0.82
verb[EXP]:lat.:scale(epoch)	0.012	0.036	0.34
case[NOM]:lat.:scale(epoch)	0.03	0.023	1.3
scale(prestim):sag.:scale(epoch)	−0.016	0.025	−0.62
verb[ALT]:sag.:scale(epoch)	−0.064	0.033	−2
verb[EXP]:sag.:scale(epoch)	−0.0083	0.037	−0.22
case[NOM]:sag.:scale(epoch)	0.042	0.024	1.8
lat.:sag.:scale(epoch)	0.0084	0.046	0.18
scale(prestim):verb[ALT]:case[NOM]:lat.	−0.0053	0.04	−0.13
scale(prestim):verb[EXP]:case[NOM]:lat.	−0.0005	0.041	−0.012
scale(prestim):verb[ALT]:case[NOM]:sag.	0.0034	0.036	0.094
scale(prestim):verb[EXP]:case[NOM]:sag.	0.019	0.037	0.52
scale(prestim):verb[ALT]:lat.:sag.	−0.046	0.078	−0.59
scale(prestim):verb[EXP]:lat.:sag.	0.019	0.081	0.23
scale(prestim):case[NOM]:lat.:sag.	−0.01	0.053	−0.19
verb[ALT]:case[NOM]:lat.:sag.	−0.016	0.067	−0.23
verb[EXP]:case[NOM]:lat.:sag.	−0.013	0.071	−0.19
scale(prestim):verb[ALT]:case[NOM]:scale(epoch)	0.0027	0.02	0.14
scale(prestim):verb[EXP]:case[NOM]:scale(epoch)	0.066	0.023	2.8
scale(prestim):verb[ALT]:lat.:scale(epoch)	−0.051	0.038	−1.3
scale(prestim):verb[EXP]:lat.:scale(epoch)	0.0041	0.044	0.092
scale(prestim):case[NOM]:lat.:scale(epoch)	−0.055	0.028	−2
verb[ALT]:case[NOM]:lat.:scale(epoch)	−0.051	0.032	−1.6
verb[EXP]:case[NOM]:lat.:scale(epoch)	0.078	0.036	2.1
scale(prestim):verb[ALT]:sag.:scale(epoch)	−0.034	0.034	−1
scale(prestim):verb[EXP]:sag.:scale(epoch)	0.1	0.04	2.5
scale(prestim):case[NOM]:sag.:scale(epoch)	−0.0066	0.025	−0.26
verb[ALT]:case[NOM]:sag.:scale(epoch)	0.016	0.033	0.49
verb[EXP]:case[NOM]:sag.:scale(epoch)	0.0076	0.037	0.2
scale(prestim):lat.:sag.:scale(epoch)	−0.048	0.054	−0.88

Continuation of Table S6 (fixed effects LPS)			
	Estimate	Std. Error	t value
verb[ALT]:lat.:sag.:scale(epoch)	−0.052	0.064	−0.81
verb[EXP]:lat.:sag.:scale(epoch)	0.058	0.073	0.79
case[NOM]:lat.:sag.:scale(epoch)	−0.025	0.046	−0.54
scale(prestim):verb[ALT]:case[NOM]:lat.:sag.	−0.029	0.078	−0.37
scale(prestim):verb[EXP]:case[NOM]:lat.:sag.	0.032	0.081	0.4
scale(prestim):verb[ALT]:case[NOM]:lat.:scale(epoch)	0.035	0.038	0.93
scale(prestim):verb[EXP]:case[NOM]:lat.:scale(epoch)	−0.035	0.044	−0.79
scale(prestim):verb[ALT]:case[NOM]:sag.:scale(epoch)	−0.037	0.034	−1.1
scale(prestim):verb[EXP]:case[NOM]:sag.:scale(epoch)	0.037	0.04	0.91
scale(prestim):verb[ALT]:lat.:sag.:scale(epoch)	0.0091	0.075	0.12
scale(prestim):verb[EXP]:lat.:sag.:scale(epoch)	−0.015	0.087	−0.17
scale(prestim):case[NOM]:lat.:sag.:scale(epoch)	0.065	0.054	1.2
verb[ALT]:case[NOM]:lat.:sag.:scale(epoch)	−5.6e−05	0.064	−0.00088
verb[EXP]:case[NOM]:lat.:sag.:scale(epoch)	0.042	0.073	0.58
scale(prestim):verb[ALT]:case[NOM]:lat.:sag.:scale(epoch)	−0.095	0.075	−1.3
scale(prestim):verb[EXP]:case[NOM]:lat.:sag.:scale(epoch)	0.0083	0.087	0.095
End of Table S6			

Table S7: Summary of residuals and random effects for model produced by the call `lmer(formula = mean ~ scale(prestim) * case * accept * lat. * sag. * scale(epoch) + (1 + case | subj) + (1 + case | item), data = filter(eeg_behav_exp, wname == "n400"), REML = FALSE, control = lmerControl(optimizer = "bobyqa", calc.derivs = TRUE))`

Linear mixed model fit by maximum likelihood

AIC	BIC	logLik	deviance	df.resid
126910	127478	-63384	126768	21879

Scaled residuals:

Min	1Q	Median	3Q	Max
-4.35	-0.62	0.04	0.65	3.42

Random effects:

Groups	Term	Std.Dev.	Corr
subj	(Intercept)	1.52599	
	case[NOM]	0.70654	0.131
item	(Intercept)	0.95151	
	case[NOM]	0.71499	-0.050
Residual		4.31136	

Number of obs: 21950, groups: subj, 23; item, 20.

Table S8: Summary of fixed effects for model produced by the call `lmer(formula = mean ~ scale(prestim) * case * accept * lat. * sag. * scale(epoch) + (1 + case | subj) + (1 + case | item), data = filter(eeg_behav_exp, wname == "n400"), REML = FALSE, control = lmerControl(optimizer = "bobyqa", calc.derivs = TRUE))`.

Beginning of Table S8 (fixed effects N400 experiencer verbs)			
	Estimate	Std. Error	t value
(Intercept)	−0.62	0.38	−1.6
scale(prestim)	−0.34	0.036	−9.5
case[NOM]	0.041	0.22	0.18
accept[0]	−0.047	0.041	−1.2
lat.	0.39	0.057	6.8
sag.	−0.83	0.059	−14
scale(epoch)	0.61	0.18	3.3
scale(prestim):case[NOM]	0.04	0.036	1.1
scale(prestim):accept[0]	−0.035	0.035	−1
case[NOM]:accept[0]	−0.021	0.041	−0.51
scale(prestim):lat.	−0.13	0.069	−1.9
case[NOM]:lat.	−0.035	0.057	−0.61
accept[0]:lat.	−0.049	0.057	−0.87
scale(prestim):sag.	0.28	0.06	4.7
case[NOM]:sag.	−0.07	0.059	−1.2
accept[0]:sag.	0.076	0.059	1.3
lat.:sag.	−0.081	0.12	−0.7
scale(prestim):scale(epoch)	−0.071	0.035	−2
case[NOM]:scale(epoch)	−0.12	0.16	−0.75
accept[0]:scale(epoch)	0.037	0.039	0.96
lat.:scale(epoch)	0.0018	0.055	0.032
sag.:scale(epoch)	0.15	0.057	2.7
scale(prestim):case[NOM]:accept[0]	0.19	0.035	5.4
scale(prestim):case[NOM]:lat.	−0.079	0.069	−1.1
scale(prestim):accept[0]:lat.	−0.01	0.069	−0.15
case[NOM]:accept[0]:lat.	−0.07	0.057	−1.2
scale(prestim):case[NOM]:sag.	−0.12	0.06	−2
scale(prestim):accept[0]:sag.	0.15	0.06	2.5
case[NOM]:accept[0]:sag.	0.13	0.059	2.3
scale(prestim):lat.:sag.	−0.065	0.13	−0.49
case[NOM]:lat.:sag.	0.05	0.12	0.43
accept[0]:lat.:sag.	0.1	0.12	0.87
scale(prestim):case[NOM]:scale(epoch)	−0.055	0.035	−1.6
scale(prestim):accept[0]:scale(epoch)	0.24	0.035	6.7
case[NOM]:accept[0]:scale(epoch)	−0.13	0.039	−3.3
scale(prestim):lat.:scale(epoch)	−0.03	0.067	−0.45

Continuation of Table S8 (fixed effects N400 experiencer verbs)			
	Estimate	Std. Error	t value
case[NOM]:lat.:scale(epoch)	−0.1	0.055	−1.9
accept[0]:lat.:scale(epoch)	−0.021	0.055	−0.39
scale(prestim):sag.:scale(epoch)	−0.048	0.061	−0.8
case[NOM]:sag.:scale(epoch)	−0.14	0.057	−2.4
accept[0]:sag.:scale(epoch)	−0.064	0.057	−1.1
lat.:sag.:scale(epoch)	0.014	0.11	0.13
scale(prestim):case[NOM]:accept[0]:lat.	−0.05	0.069	−0.72
scale(prestim):case[NOM]:accept[0]:sag.	−0.026	0.06	−0.43
scale(prestim):case[NOM]:lat.:sag.	0.023	0.13	0.17
scale(prestim):accept[0]:lat.:sag.	−0.12	0.13	−0.87
case[NOM]:accept[0]:lat.:sag.	−0.091	0.12	−0.79
scale(prestim):case[NOM]:accept[0]:scale(epoch)	−0.086	0.035	−2.4
scale(prestim):case[NOM]:lat.:scale(epoch)	0.033	0.067	0.48
scale(prestim):accept[0]:lat.:scale(epoch)	−0.1	0.067	−1.5
case[NOM]:accept[0]:lat.:scale(epoch)	−0.0082	0.055	−0.15
scale(prestim):case[NOM]:sag.:scale(epoch)	−0.031	0.061	−0.51
scale(prestim):accept[0]:sag.:scale(epoch)	0.13	0.061	2.2
case[NOM]:accept[0]:sag.:scale(epoch)	−0.14	0.057	−2.4
scale(prestim):lat.:sag.:scale(epoch)	0.034	0.13	0.26
case[NOM]:lat.:sag.:scale(epoch)	0.015	0.11	0.13
accept[0]:lat.:sag.:scale(epoch)	0.049	0.11	0.44
scale(prestim):case[NOM]:accept[0]:lat.:sag.	0.0039	0.13	0.03
scale(prestim):case[NOM]:accept[0]:lat.:scale(epoch)	0.074	0.067	1.1
scale(prestim):case[NOM]:accept[0]:sag.:scale(epoch)	0.031	0.061	0.51
scale(prestim):case[NOM]:lat.:sag.:scale(epoch)	−0.031	0.13	−0.23
scale(prestim):accept[0]:lat.:sag.:scale(epoch)	0.049	0.13	0.38
case[NOM]:accept[0]:lat.:sag.:scale(epoch)	0.006	0.11	0.054
scale(prestim):case[NOM]:accept[0]:lat.:sag.:scale(epoch)	0.00054	0.13	0.0041
End of Table S8			

Table S9: Summary of residuals and random effects for model produced by the call `lmer(formula = mean ~ scale(prestim) * case * accept * lat. * sag. * scale(epoch) + (1 + case | subj) + (1 + case | item), data = filter(eeg_behav_exp, wname == "lps"), REML = FALSE, control = lmerControl(optimizer = "bobyqa", calc.derivs = TRUE))`

Linear mixed model fit by maximum likelihood

AIC	BIC	logLik	deviance	df.resid
120799	121367	-60328	120657	21879

Scaled residuals:

Min	1Q	Median	3Q	Max
-3.86	-0.64	-0.01	0.64	4.33

Random effects:

Groups	Term	Std.Dev.	Corr
subj	(Intercept)	0.94277	
	case[NOM]	0.57831	0.042
item	(Intercept)	0.54394	
	case[NOM]	0.72052	-0.204
Residual		3.75354	

Number of obs: 21950, groups: subj, 23; item, 20.

Table S10: Summary of fixed effects for model produced by the call `lmer(formula = mean ~ scale(prestim) * case * accept * lat. * sag. * scale(epoch) + (1 + case | subj) + (1 + case | item), data = filter(eeg_behav_exp, wname == "lps"), REML = FALSE, control = lmerControl(optimizer = "bobyqa", calc.derivs = TRUE))`.

Beginning of Table S10 (fixed effects LPS experiencer verbs)			
	Estimate	Std. Error	t value
(Intercept)	0.35	0.23	1.5
scale(prestim)	−2.5	0.031	−82
case[NOM]	0.14	0.2	0.66
accept[0]	0.15	0.036	4.2
lat.	−0.15	0.05	−3.1
sag.	−0.15	0.051	−3
scale(epoch)	−0.51	0.13	−4
scale(prestim):case[NOM]	−0.07	0.031	−2.2
scale(prestim):accept[0]	−0.029	0.031	−0.93
case[NOM]:accept[0]	0.095	0.036	2.7
scale(prestim):lat.	0.12	0.06	2.1
case[NOM]:lat.	0.0041	0.05	0.083
accept[0]:lat.	−4.3e−05	0.05	−0.00087
scale(prestim):sag.	−0.18	0.052	−3.4
case[NOM]:sag.	−0.1	0.051	−2
accept[0]:sag.	−0.05	0.051	−0.97
lat.:sag.	0.26	0.1	2.6
scale(prestim):scale(epoch)	0.22	0.031	7
case[NOM]:scale(epoch)	0.16	0.14	1.2
accept[0]:scale(epoch)	0.14	0.034	4.2
lat.:scale(epoch)	0.069	0.048	1.4
sag.:scale(epoch)	−0.072	0.049	−1.5
scale(prestim):case[NOM]:accept[0]	−0.28	0.031	−9
scale(prestim):case[NOM]:lat.	0.055	0.06	0.92
scale(prestim):accept[0]:lat.	0.062	0.06	1
case[NOM]:accept[0]:lat.	0.033	0.05	0.67
scale(prestim):case[NOM]:sag.	0.094	0.052	1.8
scale(prestim):accept[0]:sag.	−0.061	0.053	−1.2
case[NOM]:accept[0]:sag.	0.0007	0.051	0.014
scale(prestim):lat.:sag.	−0.00039	0.12	−0.0034
case[NOM]:lat.:sag.	−0.018	0.1	−0.18
accept[0]:lat.:sag.	−0.11	0.1	−1.1
scale(prestim):case[NOM]:scale(epoch)	−0.011	0.031	−0.36
scale(prestim):accept[0]:scale(epoch)	−0.17	0.031	−5.4
case[NOM]:accept[0]:scale(epoch)	−0.011	0.034	−0.32
scale(prestim):lat.:scale(epoch)	0.022	0.059	0.37

Continuation of Table S10 (fixed effects LPS experiencer verbs)			
	Estimate	Std. Error	t value
case[NOM]:lat.:scale(epoch)	0.12	0.048	2.6
accept[0]:lat.:scale(epoch)	0.086	0.048	1.8
scale(prestim):sag.:scale(epoch)	0.094	0.053	1.8
case[NOM]:sag.:scale(epoch)	0.03	0.049	0.62
accept[0]:sag.:scale(epoch)	−0.069	0.049	−1.4
lat.:sag.:scale(epoch)	0.0083	0.097	0.085
scale(prestim):case[NOM]:accept[0]:lat.	0.063	0.06	1
scale(prestim):case[NOM]:accept[0]:sag.	0.076	0.053	1.5
scale(prestim):case[NOM]:lat.:sag.	0.011	0.12	0.098
scale(prestim):accept[0]:lat.:sag.	0.068	0.12	0.59
case[NOM]:accept[0]:lat.:sag.	0.024	0.1	0.24
scale(prestim):case[NOM]:accept[0]:scale(epoch)	0.06	0.031	1.9
scale(prestim):case[NOM]:lat.:scale(epoch)	−0.036	0.059	−0.61
scale(prestim):accept[0]:lat.:scale(epoch)	0.11	0.059	1.9
case[NOM]:accept[0]:lat.:scale(epoch)	0.074	0.048	1.5
scale(prestim):case[NOM]:sag.:scale(epoch)	−0.0045	0.053	−0.085
scale(prestim):accept[0]:sag.:scale(epoch)	−0.078	0.053	−1.5
case[NOM]:accept[0]:sag.:scale(epoch)	0.052	0.049	1
scale(prestim):lat.:sag.:scale(epoch)	−0.1	0.11	−0.88
case[NOM]:lat.:sag.:scale(epoch)	−0.0015	0.097	−0.016
accept[0]:lat.:sag.:scale(epoch)	−0.063	0.097	−0.65
scale(prestim):case[NOM]:accept[0]:lat.:sag.	−0.011	0.12	−0.095
scale(prestim):case[NOM]:accept[0]:lat.:scale(epoch)	0.0051	0.059	0.087
scale(prestim):case[NOM]:accept[0]:sag.:scale(epoch)	−0.005	0.053	−0.094
scale(prestim):case[NOM]:lat.:sag.:scale(epoch)	0.0068	0.11	0.06
scale(prestim):accept[0]:lat.:sag.:scale(epoch)	−0.18	0.11	−1.6
case[NOM]:accept[0]:lat.:sag.:scale(epoch)	−0.076	0.097	−0.78
scale(prestim):case[NOM]:accept[0]:lat.:sag.:scale(epoch)	−0.11	0.11	−0.96
End of Table S10			
