# Supplementary material for: Language Processing as a Precursor to Language Change: Evidence From Icelandic
Source: Front Psychol. 2020 Jan 17;10:3013. doi: 10.3389/fpsyg.2019.03013 (PMC6978737; doi:10.3389/fpsyg.2019.03013)

Supplementary Figures

Language processing as a precursor to language change: evidence from Icelandic

January 2, 2020

Figure S1: Grand average ERPs for Active verbs. Shaded regions show 95% confidence intervals by participants

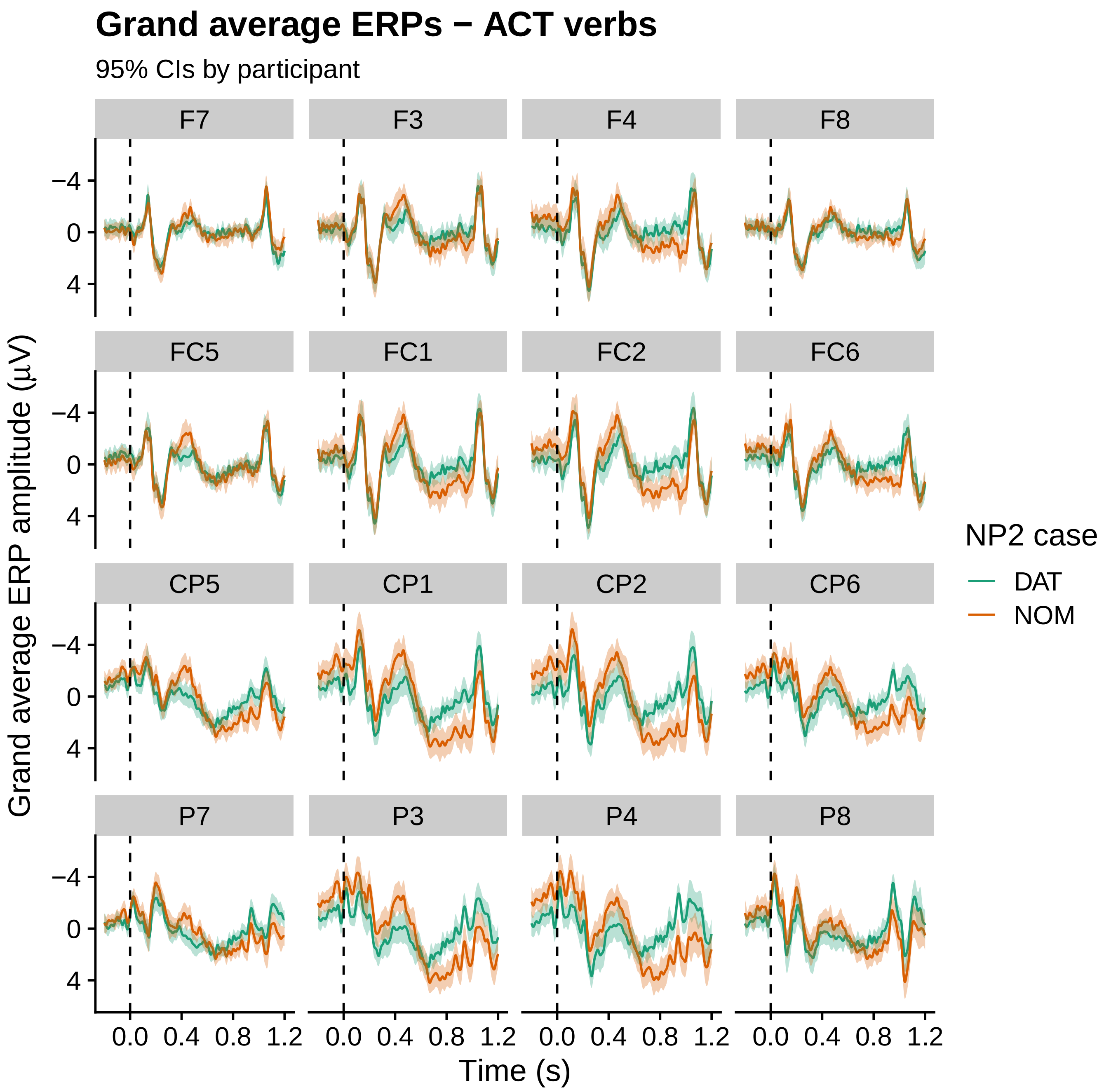

Figure S2: Grand average ERPs for Active verbs. Shaded regions show 95% confidence intervals by items

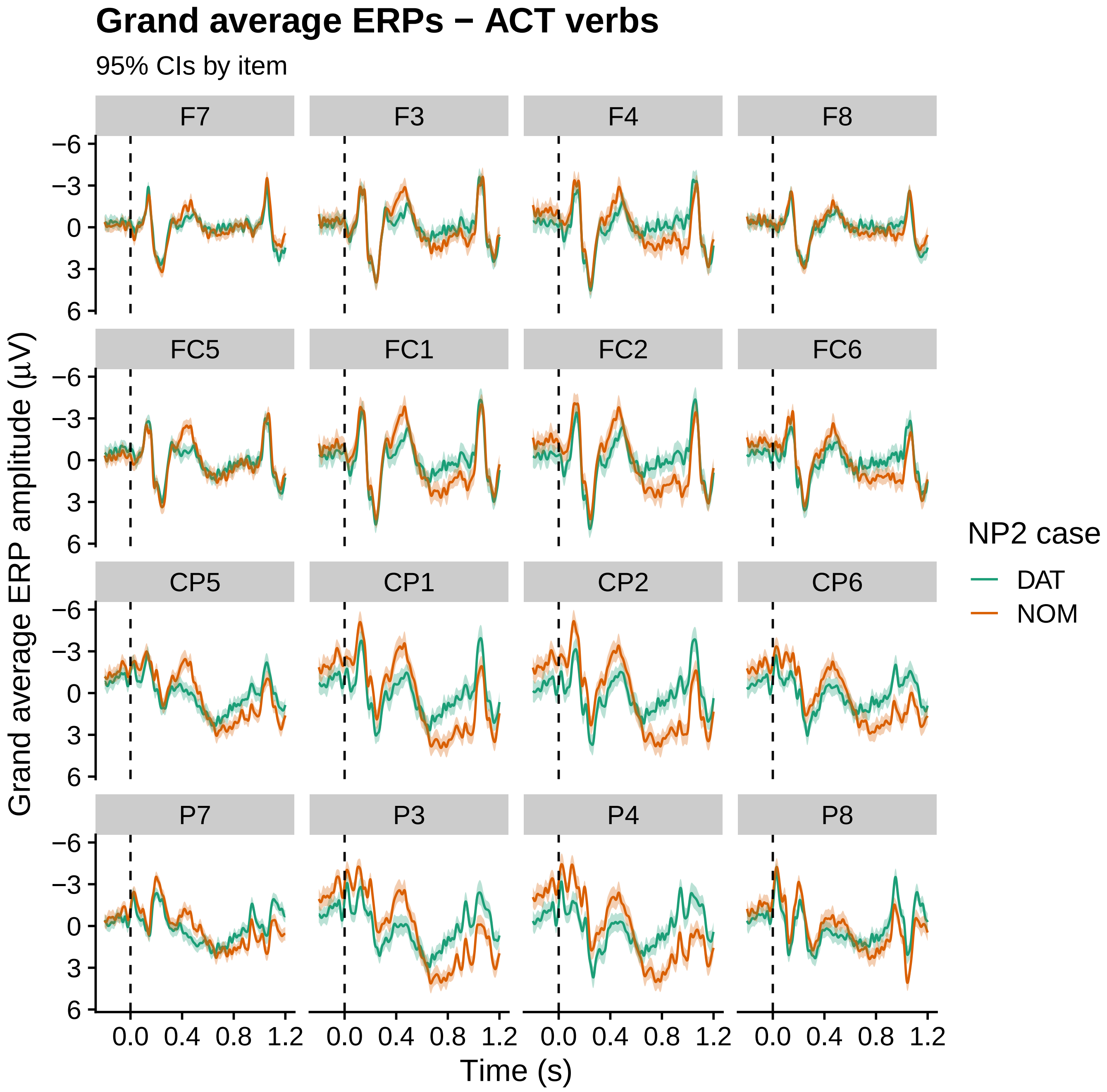

Figure S3: Grand average ERPs for Alternating verbs. Shaded regions show 95% confidence intervals by participants

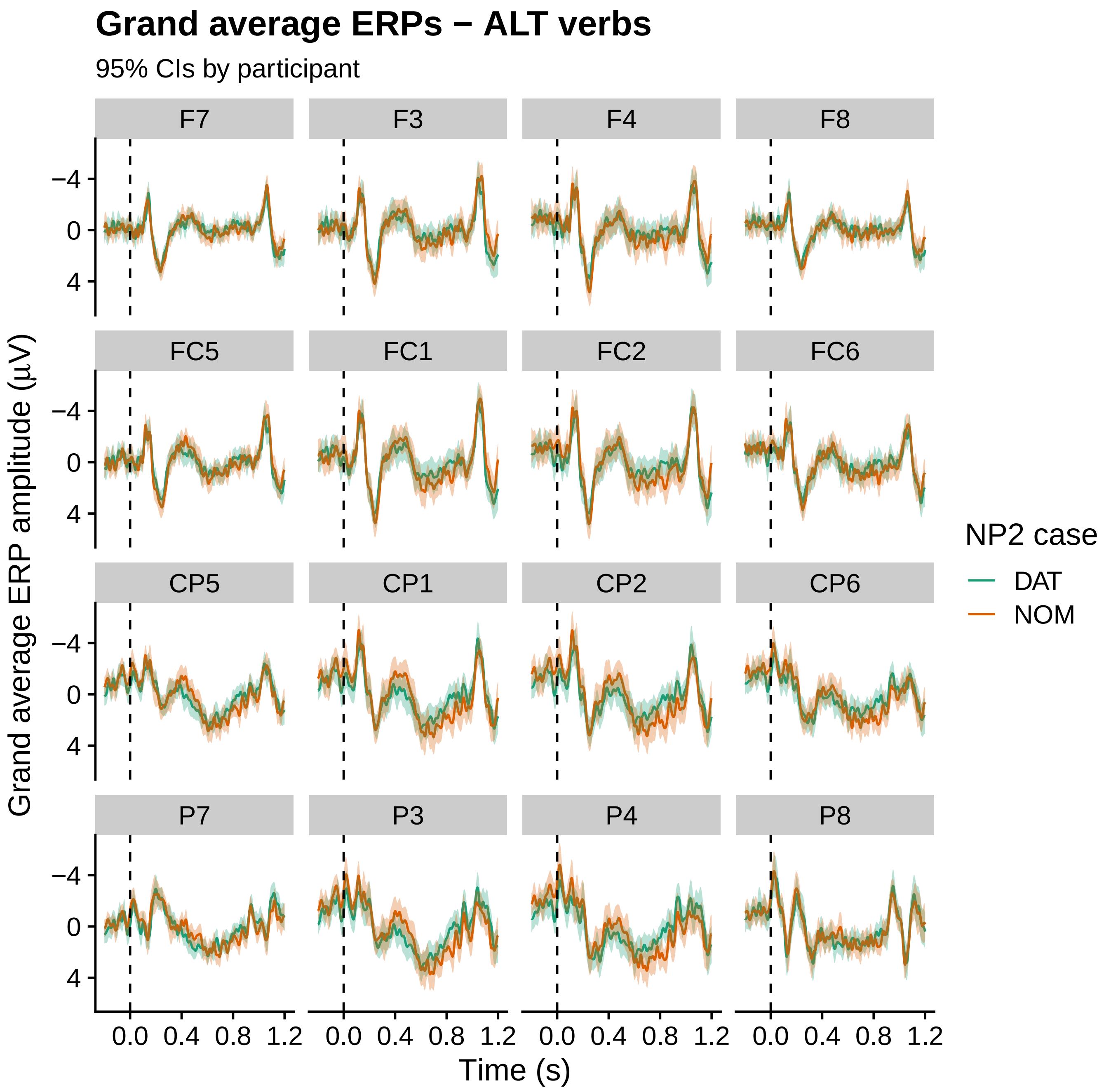

Figure S4: Grand average ERPs for Alternating verbs. Shaded regions show 95% confidence intervals by items

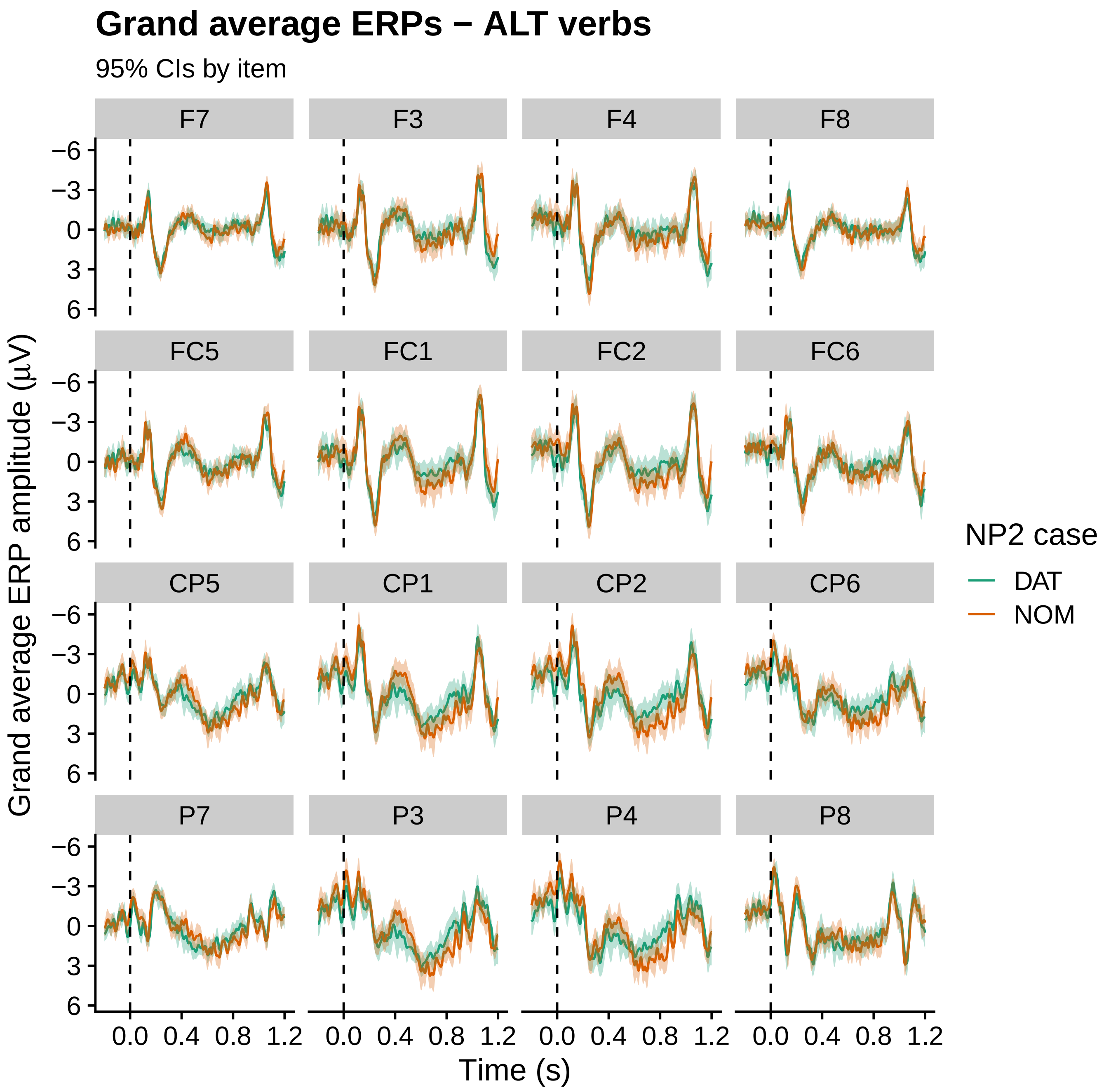

Figure S5: Grand average ERPs for Experiencer verbs. Shaded regions show 95% confidence intervals by participants

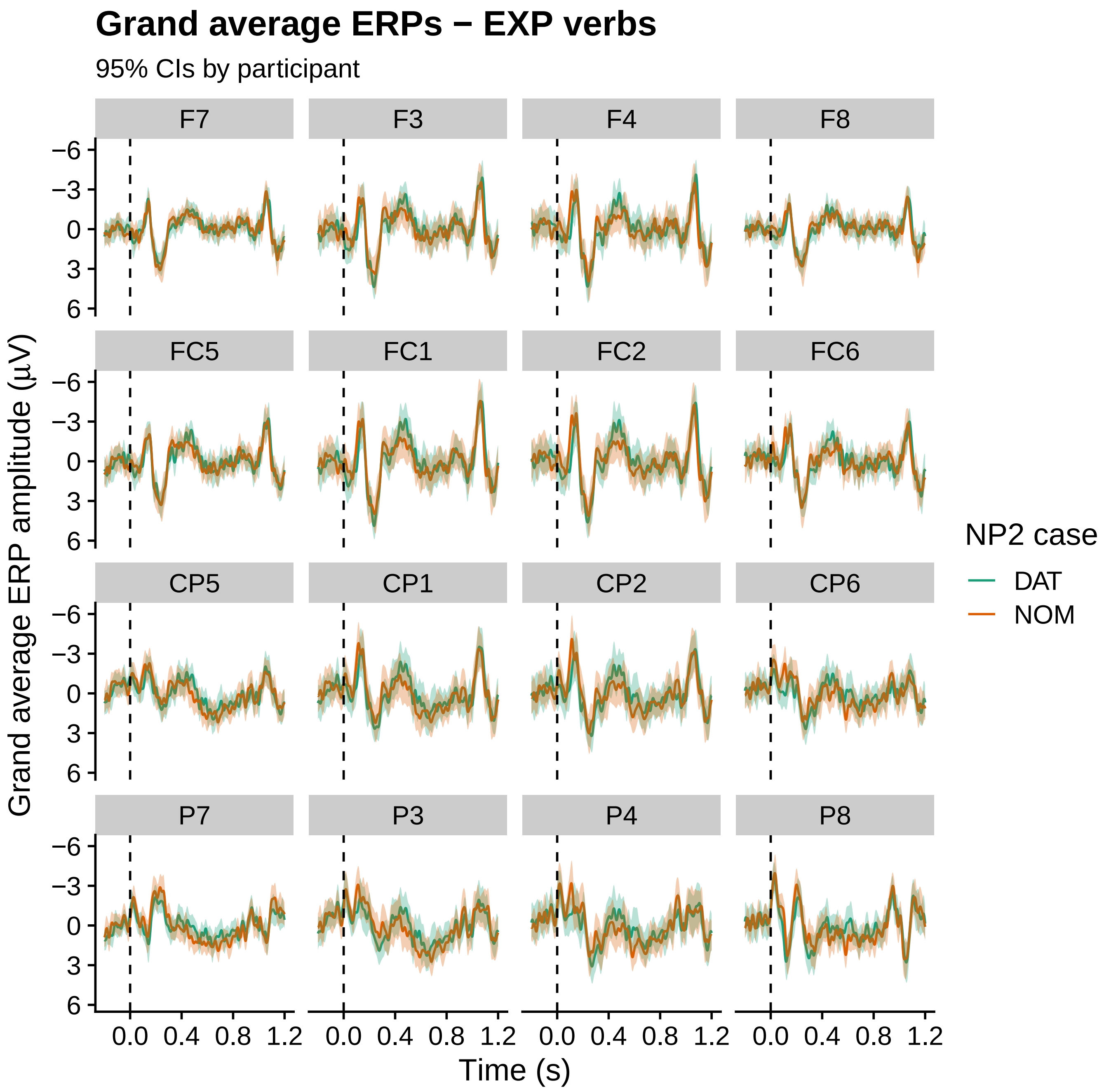

Figure S6: Grand average ERPs for Experiencer verbs. Shaded regions show 95% confidence intervals by items

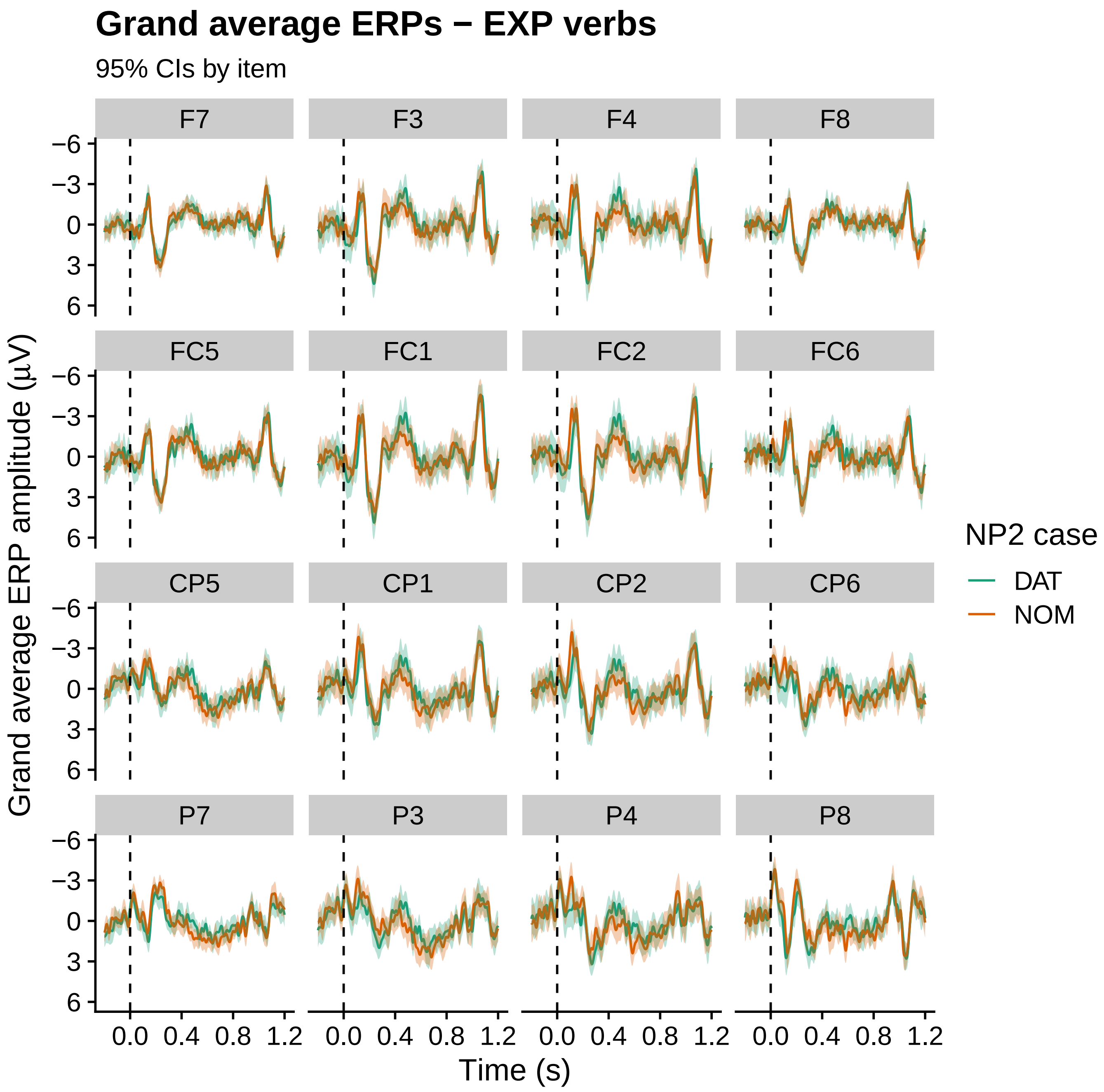

Figure S7: Resolution of verb type x case x trial x sagittality x prestimulus amplitude interaction for the N400 time window. Prestimulus amplitude = -5 μV.

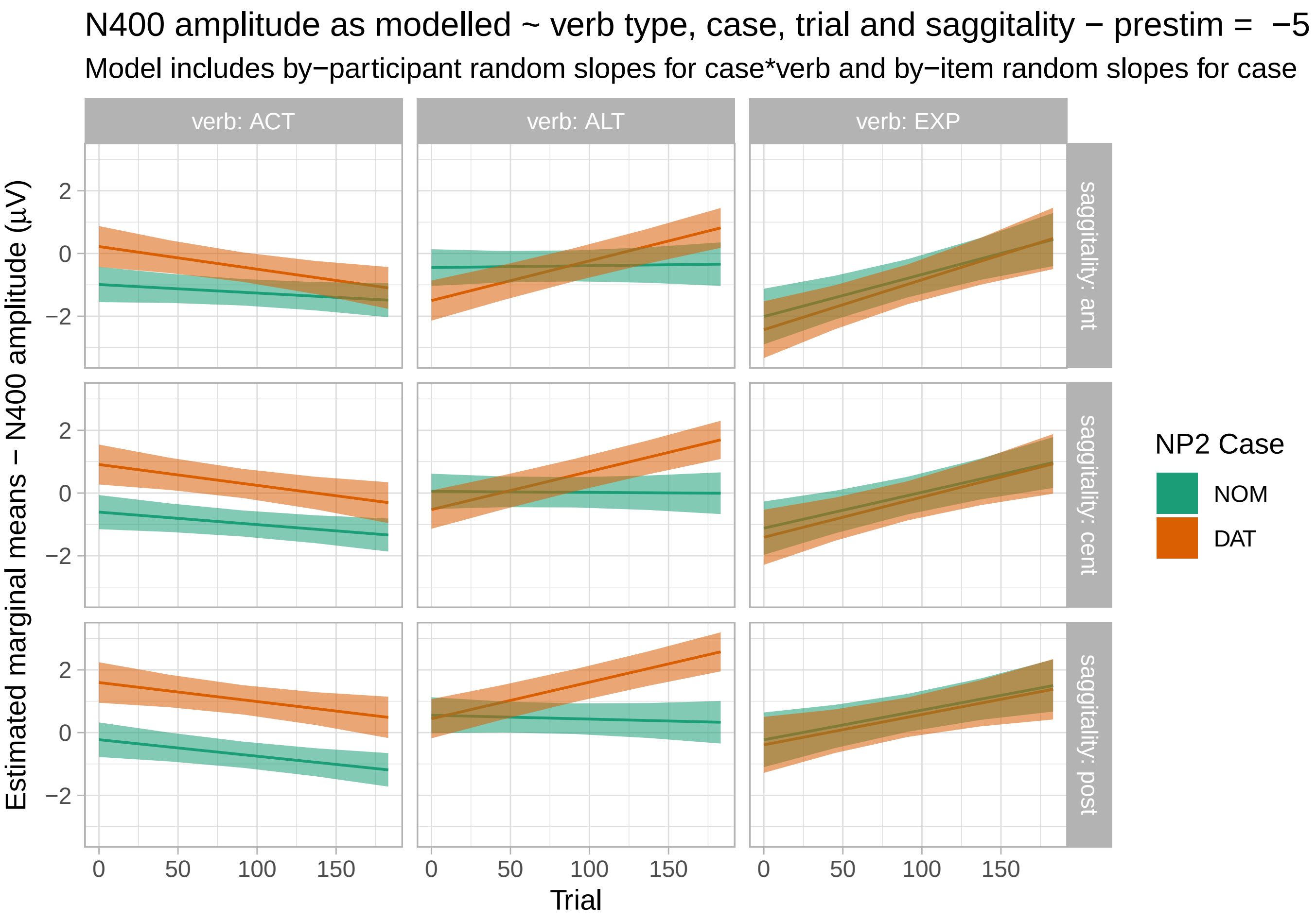

Figure S8: Resolution of verb type x case x trial x sagittality x prestimulus amplitude interaction for the N400 time window. Prestimulus amplitude = 0 μV.

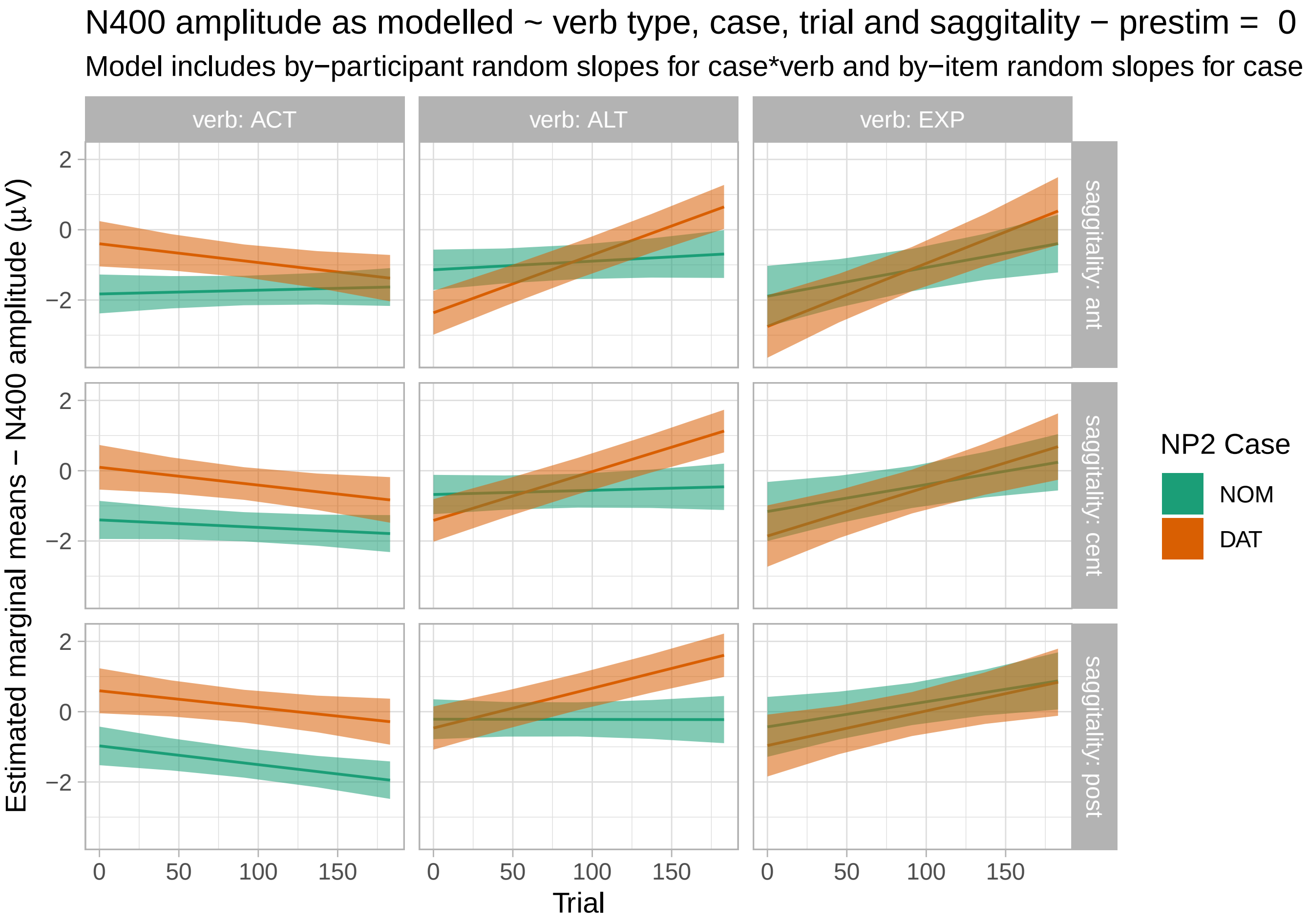

Figure S9: Resolution of verb type x case x trial x sagittality x prestimulus amplitude interaction for the N400 time window. Prestimulus amplitude = 5 μV.

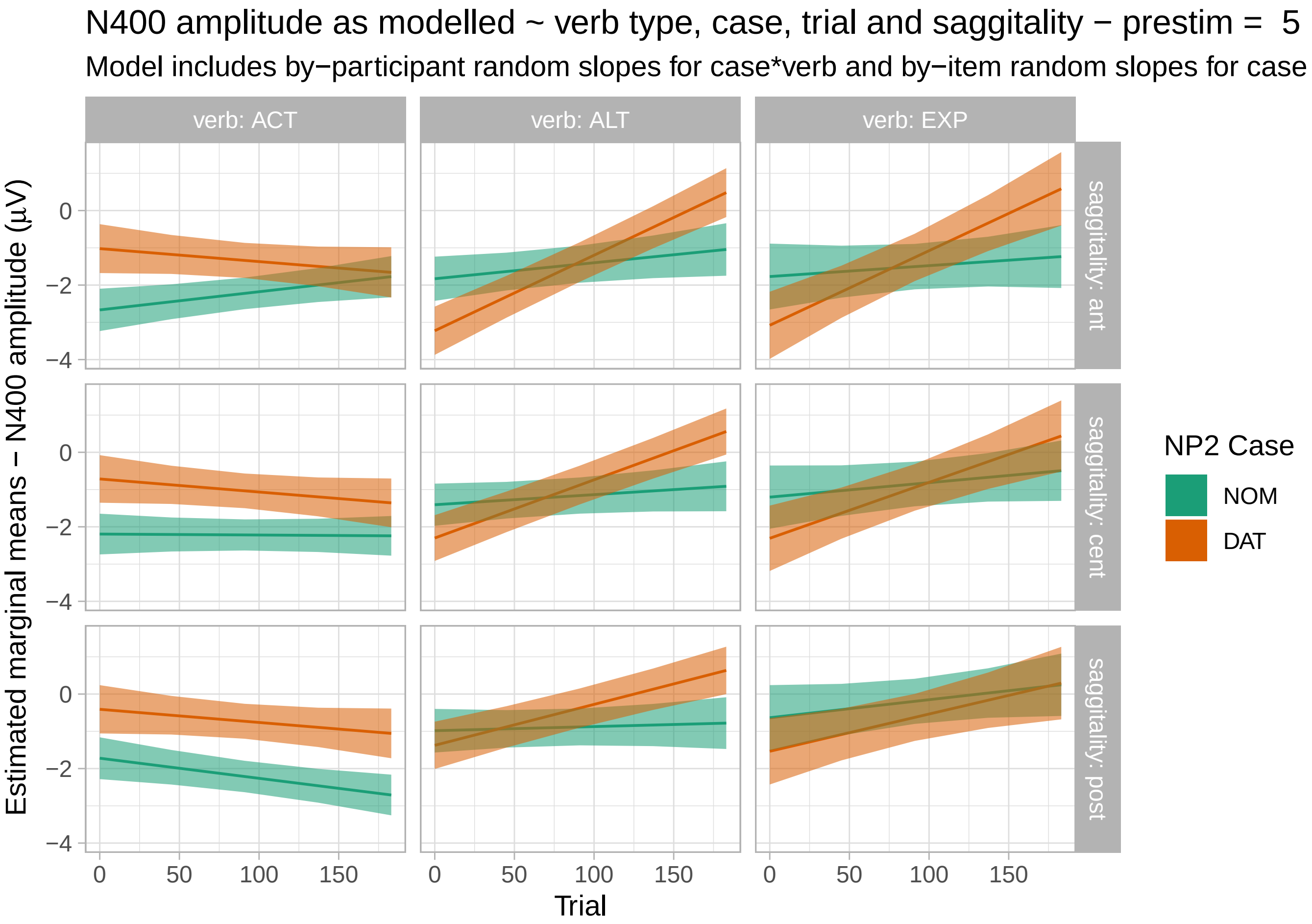

Figure S10: Resolution of verb type x case x trial x prestimulus amplitude interaction for the late positivity time window. Prestimulus amplitude = -5 μV.

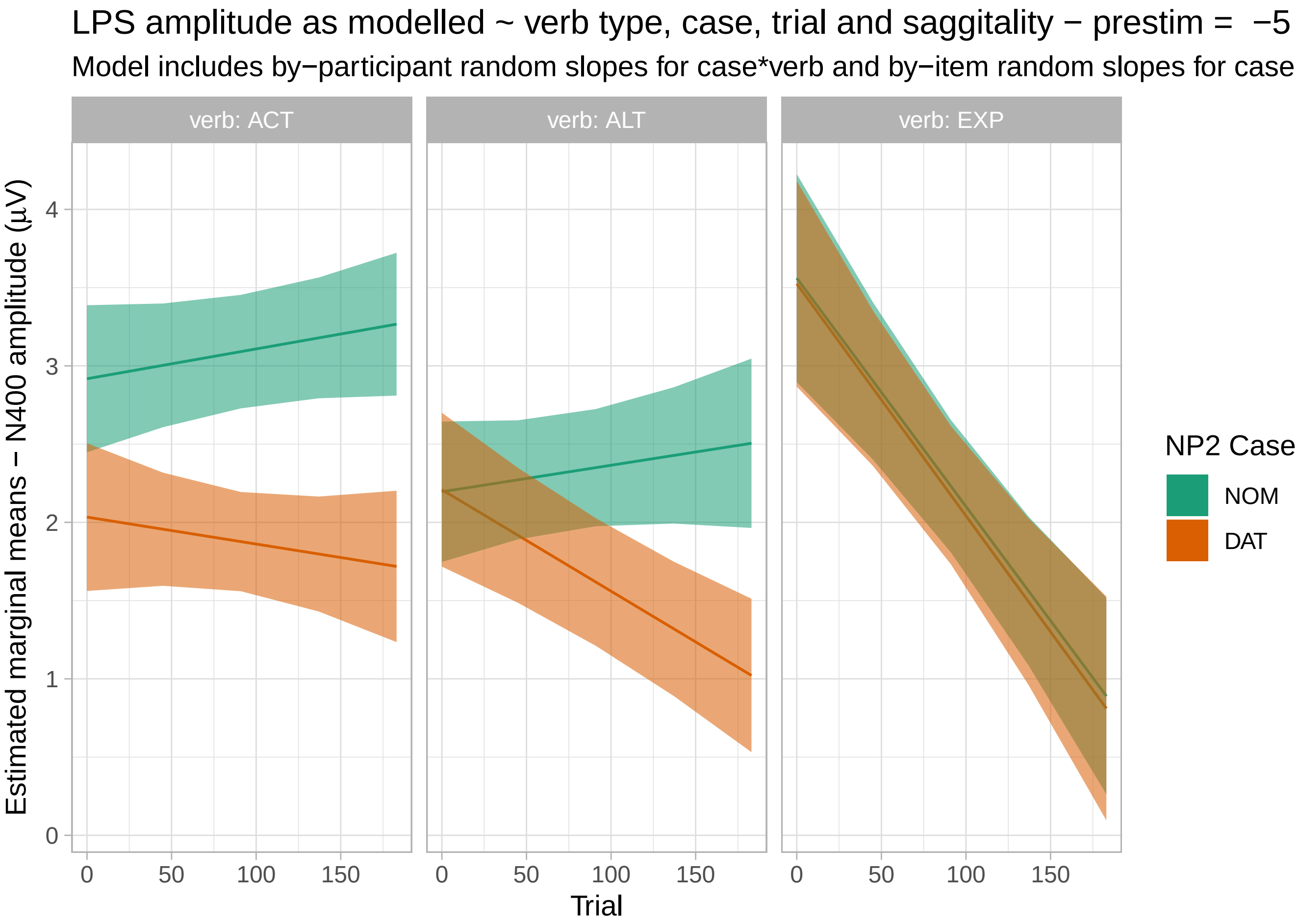

Figure S11: Resolution of verb type x case x trial x prestimulus amplitude interaction for the late positivity time window. Prestimulus amplitude = 0 μV.

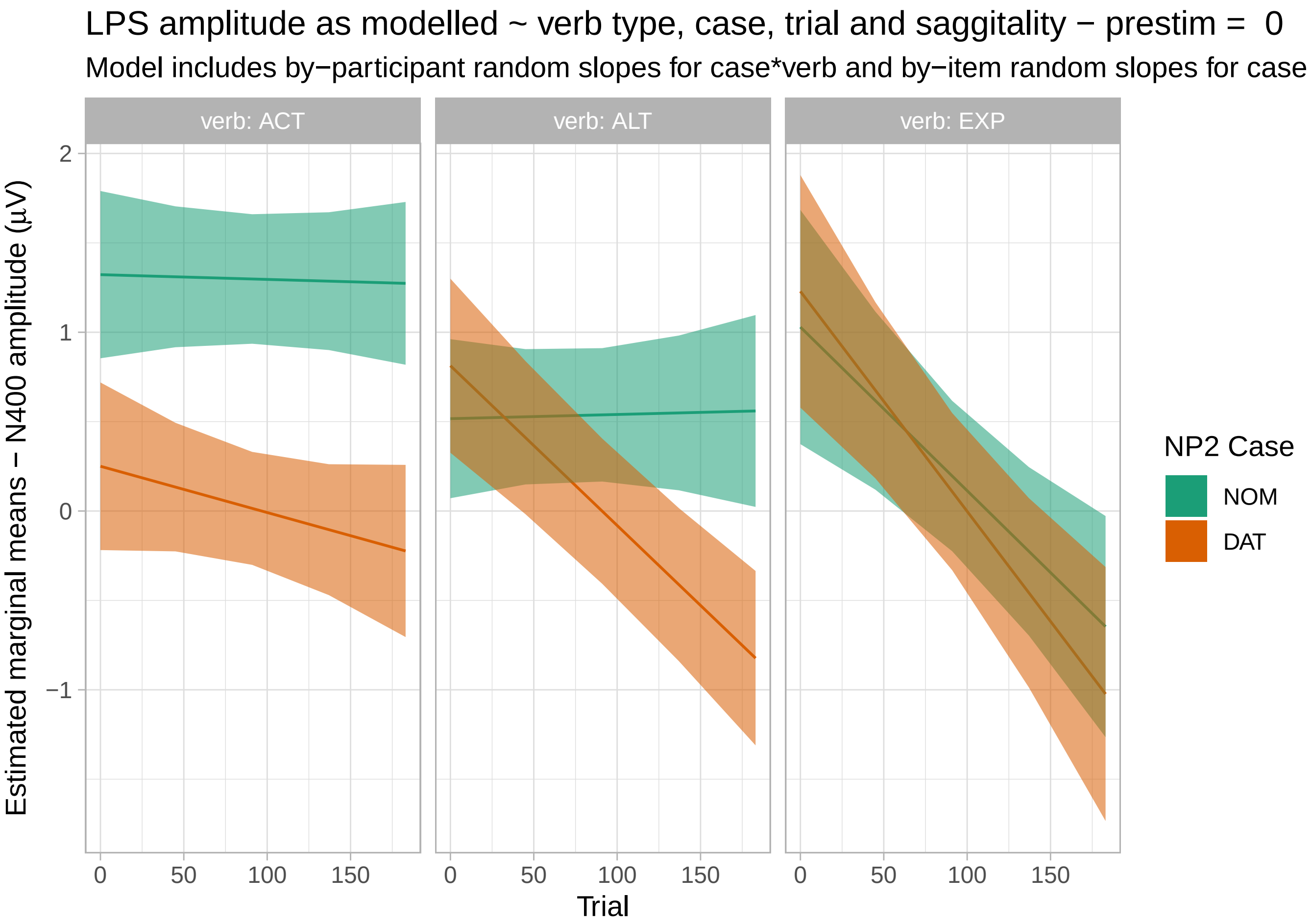

Figure S12: Resolution of verb type x case x trial x prestimulus amplitude interaction for the late positivity time window. Prestimulus amplitude = 5 μV.

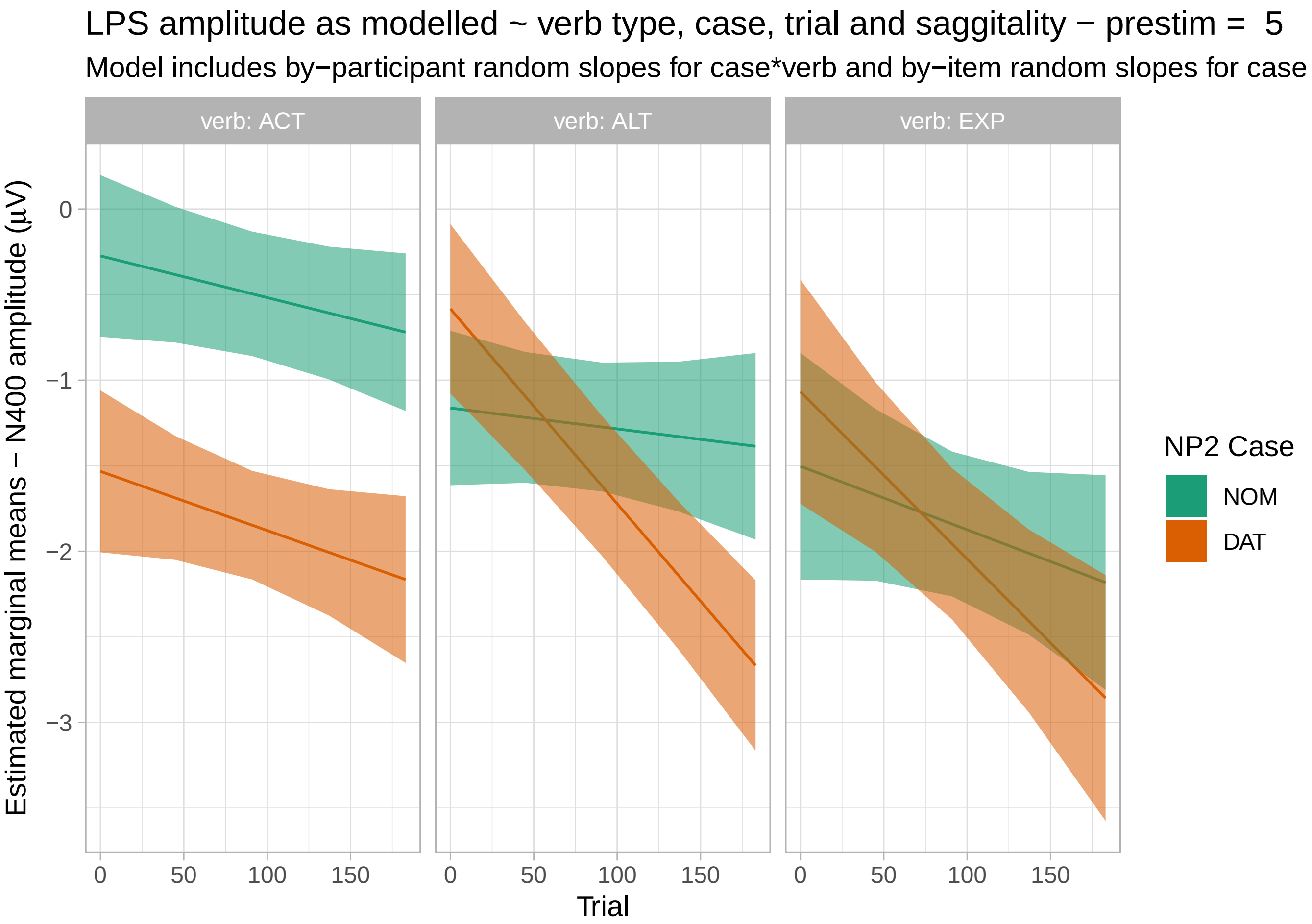

Figure S13: Resolution of case x acceptability x trial x prestimulus amplitude interaction for experiencer verbs in the late positivity time window. Prestimulus amplitude = -5 μV.

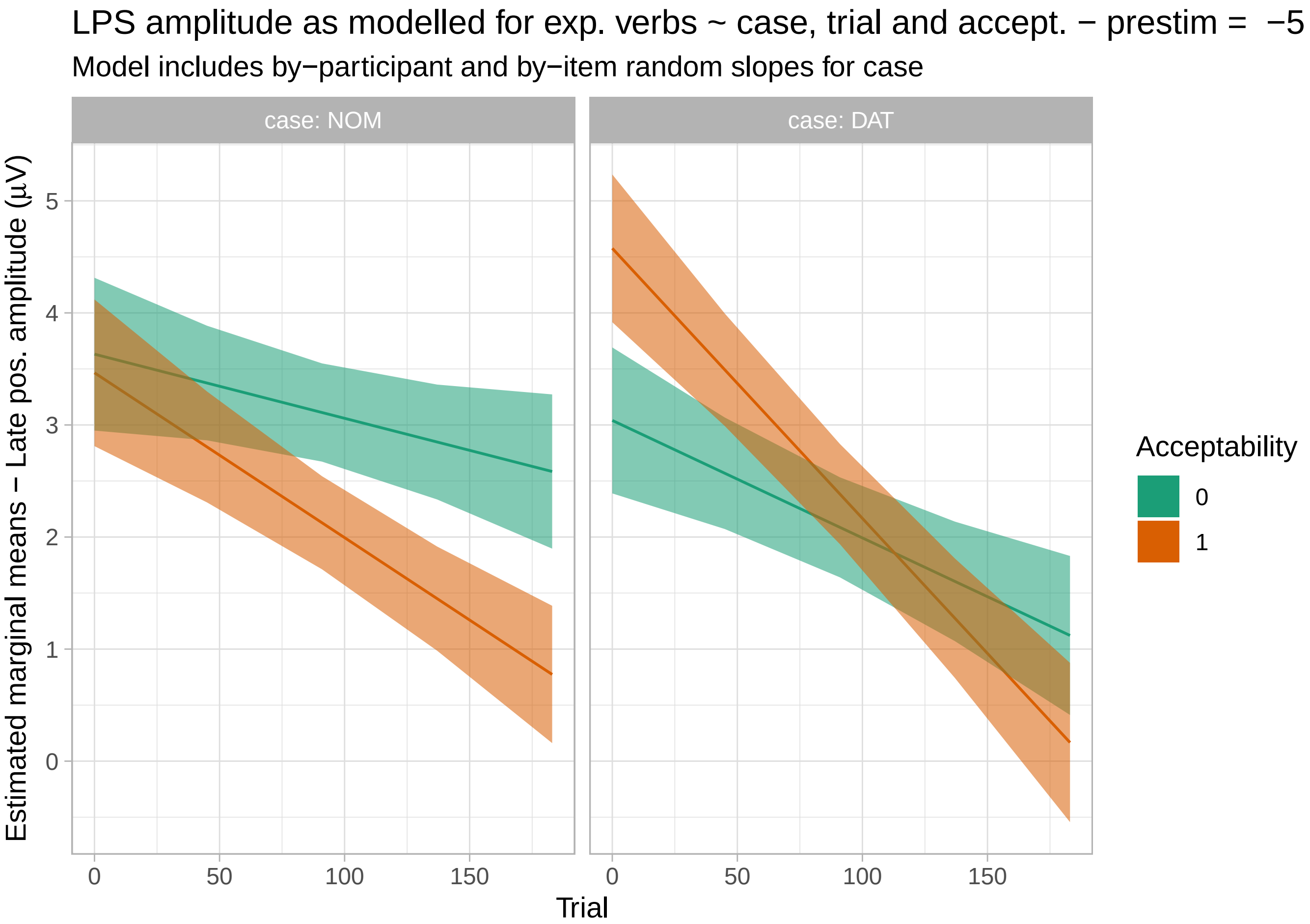

Figure S14: Resolution of case x acceptability x trial x prestimulus amplitude interaction for experiencer verbs in the late positivity time window. Prestimulus amplitude = 0 μV.

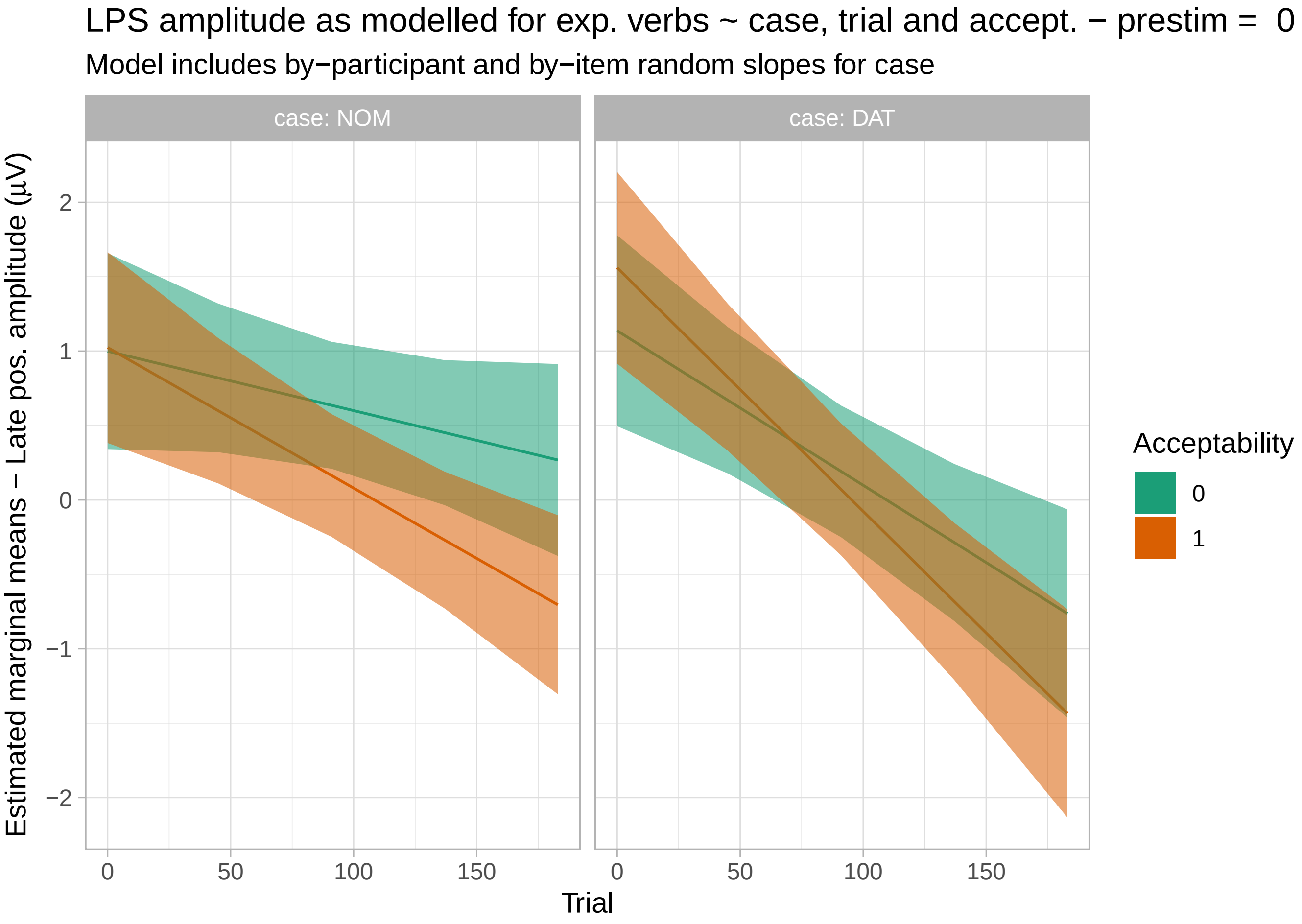

Figure S15: Resolution of case x acceptability x trial x prestimulus amplitude interaction for experiencer verbs in the late positivity time window. Prestimulus amplitude = 5 μV.

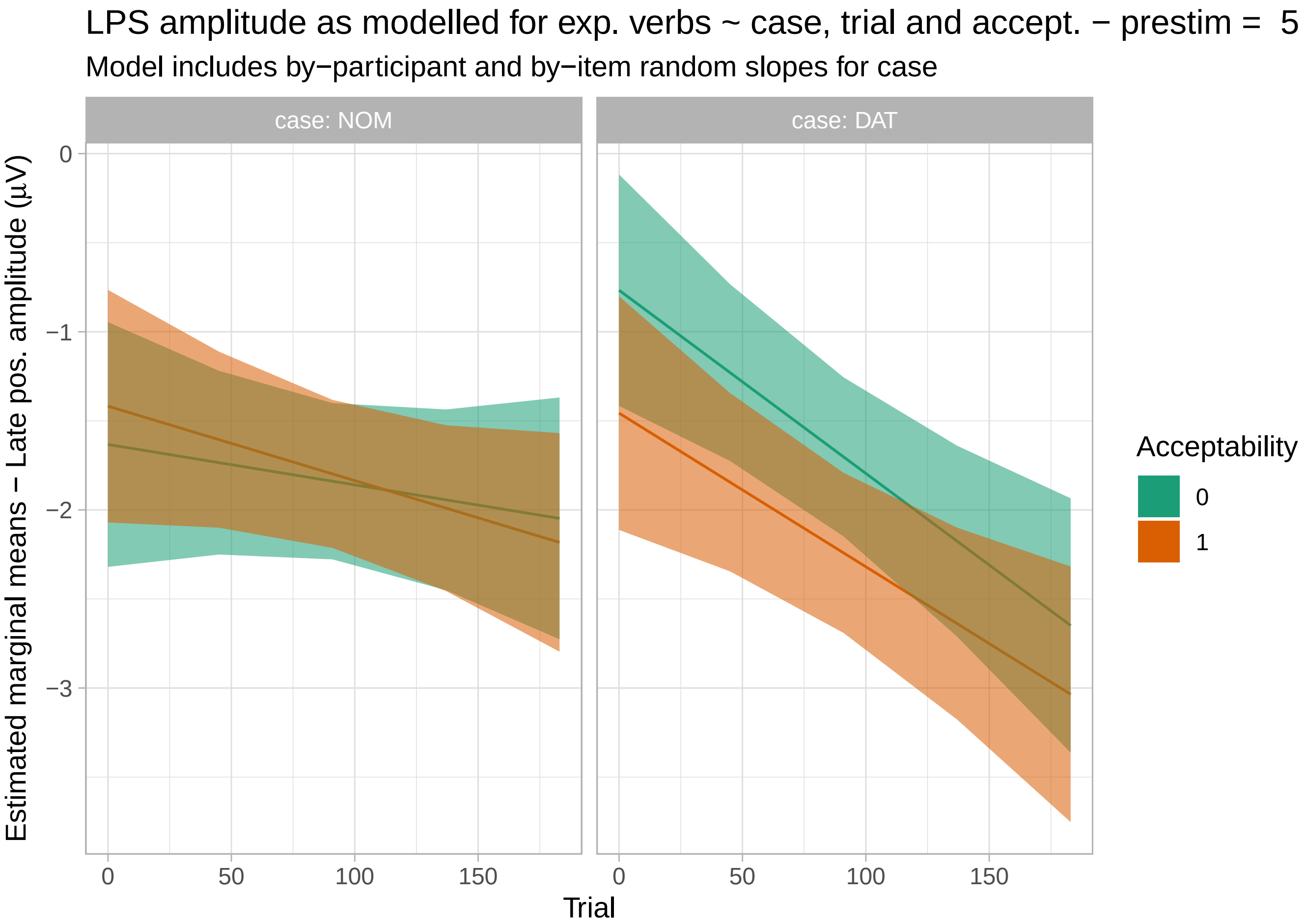

Figure S16: Trial-by-trial acceptabilities as predicted by N400 amplitude and verb type.

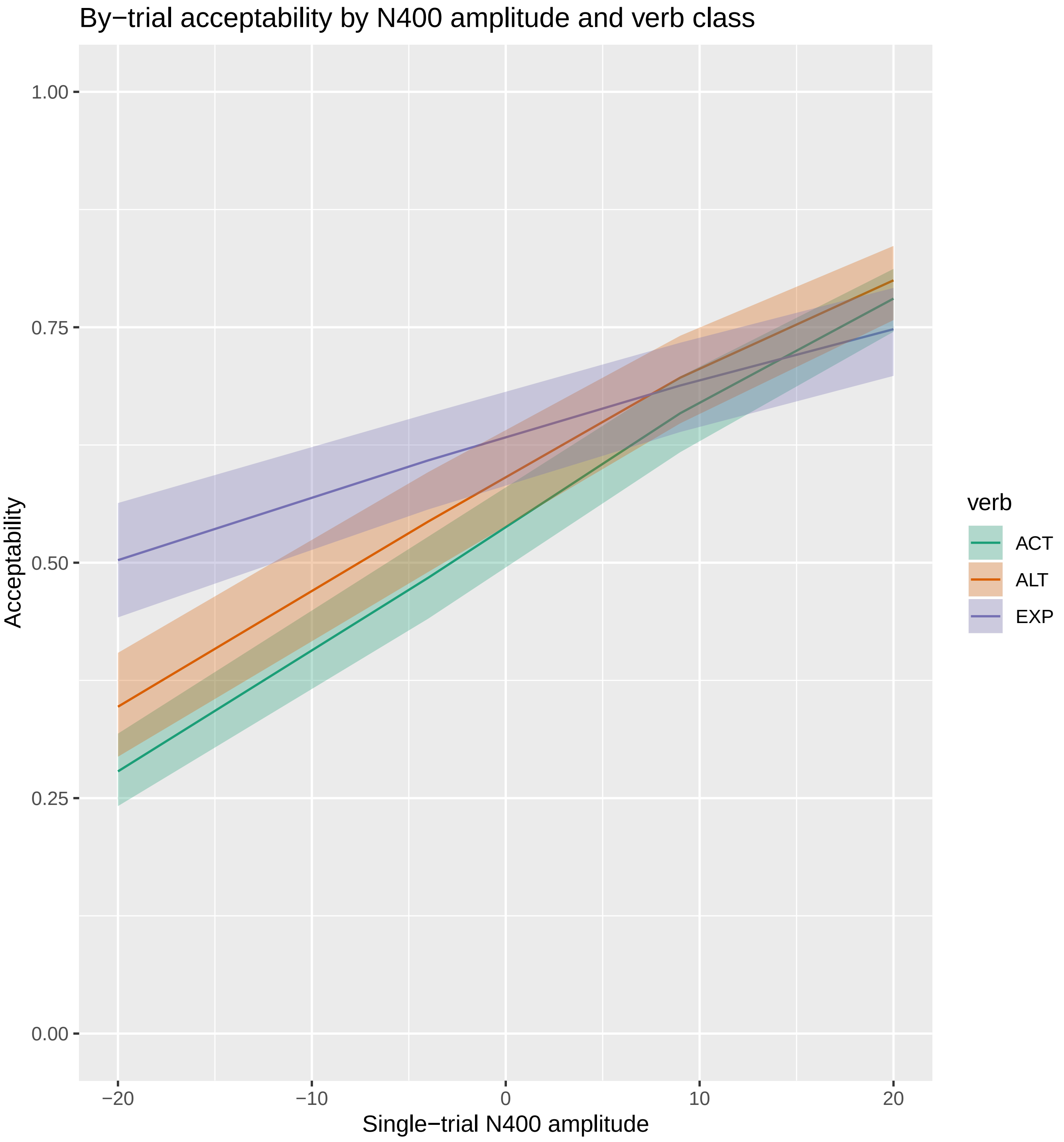

Figure S17: Trial-by-trial acceptabilities as predicted by late positivity amplitude and verb type.

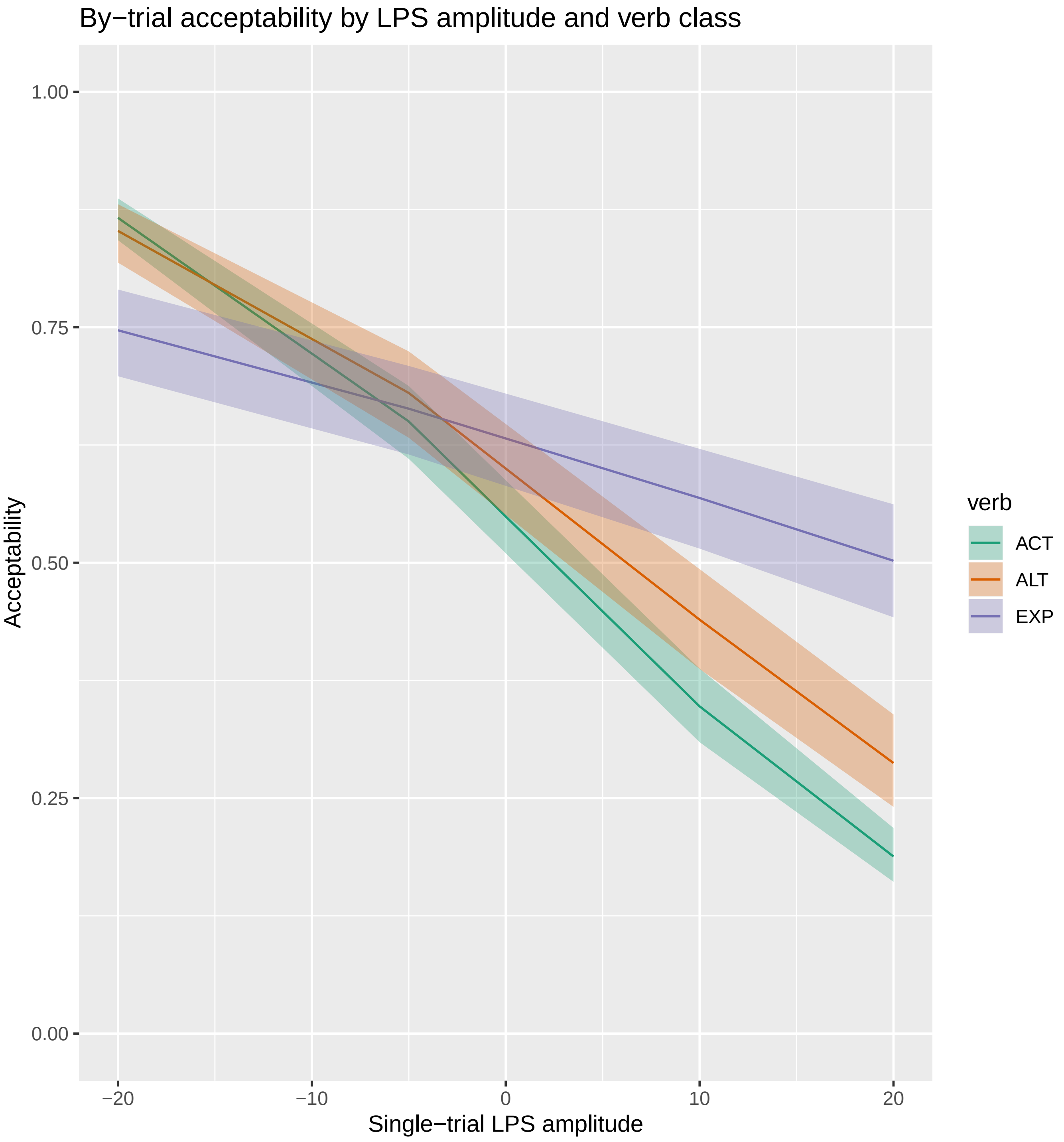

Supplement: Supplementary file 2 [file Data_Sheet_2.pdf]
